# Cloning and Characterization of *TaTGW-7A* Gene Associated with Grain Weight in Wheat via SLAF-seq-BSA

**DOI:** 10.3389/fpls.2016.01902

**Published:** 2016-12-20

**Authors:** Ming-Jian Hu, Hai-Ping Zhang, Kai Liu, Jia-Jia Cao, Sheng-Xing Wang, Hao Jiang, Zeng-Yun Wu, Jie Lu, Xiao F. Zhu, Xian-Chun Xia, Gen-Lou Sun, Chuan-Xi Ma, Cheng Chang

**Affiliations:** ^1^College of Agronomy, Anhui Agricultural University – Key Laboratory of Wheat Biology and Genetic Improvement on Southern Yellow & Huai River Valley, The Ministry of AgricultureHefei, China; ^2^National Wheat Improvement Center/The National Key Facility for Crop Gene Resources and Genetic Improvement, Institute of Crop Science, Chinese Academy of Agricultural SciencesBeijing, China; ^3^Department of Biology, Saint Mary’s University, HalifaxNS, Canada

**Keywords:** common wheat, gene expression, gene-specific marker, IAA, SLAF-seq

## Abstract

Thousand-grain weight (TGW) of wheat (*Triticum aestivum* L.) contributes significantly to grain yield. In the present study, a candidate gene associated with TGW was identified through specific-locus amplified fragment sequencing (SLAF-seq) of DNA bulks of recombinant inbred lines (RIL) derived from the cross between Jing 411 and Hongmangchun 21. The gene was located on chromosome 7A, designated as *TaTGW-7A* with a complete genome sequence and an open reading frame (ORF). A single nucleotide polymorphism (SNP) was present in the first exon between two alleles at *TaTGW-7A* locus, resulting in a Val to Ala substitution, corresponding to a change from higher to lower TGW. Cleaved amplified polymorphic sequence (CAPS) (*TGW7A*) and InDel (*TG9*) markers were developed to discriminate the two alleles *TaTGW-7Aa* and *TaTGW-7Ab* for higher and lower TGW, respectively. A major QTL co-segregating with *TaTGW-7A* explained 21.7–27.1% of phenotypic variance for TGW in the RIL population across five environments. The association of *TaTGW-7A* with TGW was further validated in a natural population and Chinese mini-core collections. Quantitative real-time PCR revealed higher transcript levels of *TaTGW-7Aa* than those of *TaTGW-7Ab* during grain development. High frequencies of the superior allele *TaTGW-7Aa* for higher TGW in Chinese mini-core collections (65.0%) and 501 wheat varieties (86.0%) indicated a strong and positive selection of this allele in wheat breeding. The molecular markers *TGW7A* and *TG9* can be used for improvement of TGW in breeding programs.

## Introduction

Thousand-grain weight is the most stable component of wheat grain yield (Giura and Saulescu, 1996). Improvement of TGW is one of the main targets in wheat breeding. Previous studies showed that wheat TGW is controlled by polygenes (Giura and Saulescu, 1996; Huang et al., 2003; Kuchel et al., 2007). Many QTL for TGW have been identified on almost all wheat chromosomes (Huang et al., 2003, 2006; Quarrie et al., 2005; Breseghello and Sorrells, 2006; Sun et al., 2009; Lopes et al., 2013; Simmonds et al., 2014; Su et al., 2016). However, only a few major QTL have been cloned due to large genome size and limited genome sequence information in wheat. The homology-based cloning of wheat genes is a common approach based on the collinearity in cereal crops (Choi et al., 2004; Gupta et al., 2008; Hu et al., 2016). *TaGW2*, encoding an E3 ubiquitin ligase and associated with GW and TGW, was cloned based on the homologous rice gene *GW2* (Su et al., 2011). Similarly, *TaCwi-A1* associated with TGW (Ma et al., 2012; Jiang et al., 2015), *TaGASR7-A1* for GL and yield (Dong et al., 2014), *TaGS-D1* for GL and TGW (Zhang et al., 2014), and *TaTGW6* encoding an indole-3-acetic acid (IAA)-glucose hydrolase (Hu et al., 2016) have been cloned based on rice gene sequences.

Homology-based cloning is an efficient approach for isolation of wheat genes following known rice gene sequences. But obviously, it is not feasible for identification of unknown functional genes. The genetic network controlling TGW is very complex, and the molecular regulatory mechanisms may differ among diverse germplasm resources (Browne et al., 2006). Identification of more TGW genes will not only accelerate multi-gene pyramiding in wheat breeding, but it is also important for further elucidating the molecular mechanism of yield formation.

Specific-locus amplified fragment sequencing (SLAF-seq) is a high-throughput strategy for a large scale of SNP discovery and genotyping based on next generation sequencing (Sun et al., 2013). Bulked segregant analysis (BSA) is an efficient approach to detect major QTL by genotyping two DNA bulks from artificial population lines with contrasting phenotypes (Quarrie et al., 1999; Pomraning et al., 2011). Candidate genes for targeting traits can be identified rapidly using super-BSA. This approach was successfully used in detection of QTL, fine mapping of candidate genes and development of molecular markers in high plants (Trick et al., 2012; Qin et al., 2015; Xu et al., 2015a,b). In our previous studies, several genes for TGW, including *TaCKOX6a02* (Lu et al., 2015), *Tackx4* (Chang et al., 2015), and *TaTGW6* (Hu et al., 2016), have been identified using RIL from a cross between Jing 411 and Hongmangchun 21. The two parents, Jing 411 and Hongmangchun 21, showed large differences in grain size, TGW, and grain filling rate. In this study, therefore, the objectives were to (i) use super-BSA and bio-information analysis to identify candidate genes for TGW, (ii) and develop functional markers for marker-assisted selection in wheat breeding.

## Materials and Methods

### Plant Materials and Field Trials

Jing 411 is a wheat variety with high TGW (47.6 g on average over five cropping seasons); while Hongmangchun 21 is a wheat landrace with low TGW (19.7 g on average over five cropping seasons). An F_8_ RIL population (Pop 1, 150 lines kindly provided by Prof. Shihe Xiao at Chinese Academy of Agricultural Sciences) derived from the Jing 411 × Hongmangchun 21 cross was used for candidate gene mapping (**Supplementary Table S1**). Two hundred and forty-four wheat varieties, including 17 Chinese landraces and 227 modern varieties (Pop 2, **Supplementary Table S2**), and 257 accessions from Chinese wheat mini-core collections (Pop 3, kindly provided by Prof. Jizeng Jia at Chinese Academy of Agricultural Sciences) were used to validate the gene-specific marker (Hao et al., 2008) (**Supplementary Table S3**). Five hundred and one wheat varieties, including historical varieties, modern varieties, advanced lines and landraces, collected from seven wheat regions of China (Pop 4, **Supplementary Table S4**), and 180 varieties with detailed information of origins among Chinese wheat mini-core collections mentioned above were used for characterizing allelic distributions of target gene. The Pop 1 was grown at the Experimental Station of Anhui Agricultural University (Hefei, Anhui, China: 31°58′N, 117°240′E) in 2007–2008, 2010–2011, 2011–2012, 2013–2014, and 2014–2015 cropping seasons; Pop 2 was planted in 2011–2012, 2012–2013, and 2013–2014 cropping seasons, and Pop 3 in 2014–2015 cropping season. Field trials were conducted in randomized complete blocks with two replications, with double 4-m rows spaced 25 cm apart. Field management followed local agricultural practice. The flowering time was recorded for each line.

### Grain Size and Thousand Grain Weight Assay

The TGW was evaluated by weighing 1000 grains in triplicate each plot and the averaged data were used for subsequent analysis. Three hundred grains were measured to get average GL and GW with the SC-G wheat grain appearance quality image analysis system developed by the Hangzhou WSeen Detection Technology Co. Ltd., Hangzhou, China (Yin et al., 2015).

### DNA and RNA Extraction

Genomic DNA was extracted from seeds following Kang et al. (1998). RNA was isolated from seeds of six low- and six high-TGW wheat varieties using a MiniBEST Plant RNA Extraction Kit (Takara). Immature grains collected at 10 days, 20 days, and 30 DPA, and mature seeds were used in these analyses. The cDNA was synthesized using Promega GoScript (Promega, Beijing, China) according to the manufacturer’s instructions.

### SLAF Library Construction and High-Throughput Sequencing

Two DNA bulks of RILs used for BSA were formed from 30 high- and 30 low-TGW lines, respectively. The SLAF library was constructed following previous studies (Trick et al., 2012; Sun et al., 2013; Xu et al., 2015b) with minor modifications. In brief, we used *Triticum aestivum* as reference genome for a preliminary SLAF experiment to determine conditions and appropriate restriction enzymes. Genomic DNA of two bulks and parents were incubated with *RsaI* (New England Biolabs, Nanjing, China). A single-nucleotide (A) overhang was added to the digested fragments with a Klenow Fragment (3′→5′ exo-) (New England Biolabs, Nanjing, China) and dATP at 37°C, then PAGE-purified duplex tag-labeled sequencing adapters (Life Technologies, Gaithersburg, MD, USA) were ligated to the A-tailed DNA with T4 DNA ligase. PCR was performed using diluted restriction-ligation samples, dNTP, Q5^®^ High-Fidelity DNA Polymerase, and PAGE-purified PCR primers 5′-AATGATACGGCGACCACCGA-3′ and 5′-CAAGCAGAAGACGGCATACG-3′ (Life Technologies, Gaithersburg, MD, USA).

The PCR products were purified using Agencourt AMPure XP beads (Beckman Coulter, High Wycombe, UK) and pooled. The pooled sample was separated by electrophoresis on a 2.0% agarose gel. Fragments with 464–484 bp were excised, and purified using a QIAquick Gel Extraction Kit (Qiagen, Beijing, China). The gel-purified products were sequenced using the Illumina HiSeq^TM^ 2500 platform (Illumina Inc., San Diego, CA, USA) according to the manufacturer’s recommendations by the Biomarker Technologies Co, LTD (Beijing, China^1^).

### SLAF Analysis and Gene Prediction

The SLAF-seq data were processed as described by Sun et al. (2013). In brief, low-quality reads with a quality score <Q30 (means a sequencing quality score of 30, indicating a 0.1% chance of error, and 99.9% confidence) were filtered out. In the present study, SLAFs from two parents with sequencing depth <5X were removed. Additionally, the polymorphic SLAFs with more than three SNP were also filtered out. The rest polymorphic SLAF tags were clustered and mapped onto the wheat reference genome based on similarity using BLAT and short oligonucleotide alignment program (SOAP) (Kent, 2002; Li et al., 2008). Sequences with more than 90% similarity were grouped into one SLAF locus. The sequence error rate was estimated using *Arabidopsis thaliana* genome sequence data as the control (Davey et al., 2013). Finally, the SLAF-tag with same genotype as female parent (Jing 411) or the male (Hongmangchun 21) was considered as a SLAF marker and used for subsequent association analysis.

Association analysis was conducted using the SNP_index method as described by Hill et al. (2013). The Δ(SNP_index) was calculated according to Hill et al. (2013) and Xu et al. (2015c), Δ(SNP_index) value was calculated as follows: SNP_index (ab) = Mab/(Pab+Mab), SNP_index(aa) = Maa/(Paa+Maa), and Δ(SNP_index) = SNP_index (aa)-SNP_index (ab); where Maa and Mab mean the depth of aa and ab populations derived from M, respectively, Paa and Pab mean the depth of aa and ab populations derived from P, respectively. The closer marker is associated with phenotype while the closer or equal Δ(SNP_index) is to 1. The threshold value of associated SLAFs was determined when the value ≥Δ(SNP_index) of 99.99% of SLAFs. In this study, the threshold was 1 based on the analysis of **Supplementary Table S5** (Hill et al., 2013; Xu et al., 2015c). Association region with three or more consecutive polymorphic SLAF tags with Δ(SNP_index) = 1 was identified as a hot region related to TGW on wheat chromosome (Zhang et al., 2015).

The sequences of tags highly significantly associated with target traits in hot regions were extended 50 kb forward and reverse according to the sequences’ information of wheat BAC library of the Biomarker Technologies Co, LTD (Beijing, China^1^) and international GenBank including NCBI^2^, Wheat-urgi^3^, and Cerealsdb^4^. And then, these extended sequences were used for functional annotation and gene/transcript set/pathway enrichment analyses, based on the wheat genome information (Kanehisa, 2002; Gene Ontology Consortium, 2004; Subramanian et al., 2005; Brenchley et al., 2012; Zhang et al., 2015, 2016). Meanwhile, these SLAFs were also used for BLAST^3^ to obtain the homology sequences in wheat.

### Candidate Gene Cloning and Development of CAPS and InDel Markers

Eight sets of primer pairs were designed using Primer Premier 5 software based on the sequence of 7AS_4248784 and its transcript Traes_7AS_378A12AA9.1 on 7AS, which were obtained by BLAST and functional annotation and gene/transcript set/pathway enrichment analyses of SLAF65386 at a hot region on chromosome 7A. These primer pairs were used for amplifying genomic DNA and cDNA sequences of candidate gene, as well as developing InDel and CAPS markers (**Supplementary Table S6**).

The PCR reactions (total volume, 10 μL) included 0.25 μM each primer, 0.25 mM dNTP, 0.5 unit LA *Taq*, 1 μL 10 × LA PCR buffer (Takara, Dalian, China) and 100 ng of genomic DNA. The PCR conditions were 95°C for 5 min, followed by 36 cycles of 95°C for 45 s, annealing (55–62 °C) for 50 s, and extension at 72°C for 1 min, with a final extension at 72°C for 12 min. Amplified PCR fragments were separated on a 6% denaturing polyacrylamide gel or a 1.5% agarose gel. For CAPS markers, PCR products were digested with *BsmAI* (NEB), *BstNI, MluCI*, or *AluI* according to the manufacturer’s directions, labelled with fluorochrome, and then, detected on a 2.0% agarose gel.

For candidate gene cloning, the targeting PCR fragments were recovered and cloned into the pEASY-T5 simple vector and transformed into T1 competent cells (TransGen Biotech, Beijing, China). Targeting gene sequencing was conducted in an Applied Biosystems 3500 genetic analyzer (Applied Biosystems, Shanghai, China) according to the manufacturer’s instructions. Sequence alignments among different wheat varieties were performed using DNAMAN 6.0^5^. The core elements of promoter were predicted by the TSSP program^6^.

### Validation of Gene Effect by QTL and Association Analysis

QTL mapping for TGW was performed with SSR markers in Pop 1 (Somers et al., 2004^7^). The polymorphic markers between two parents and bulks were used for genotyping the entire Pop 1. The effect of candidate gene on grain weight was estimated by QTL analysis using composite interval mapping (QTL IciMapping v.4.0 software^8^). QTL was declared as significant in a LOD threshold of 3.0. As for *TaTGW-7A* linkage analysis with 61 SSR markers, two gene-specific markers and four CAPS markers on 7A, the LOD value is 5.0.

CAPS (*TGW7A*) and InDel (*TG9*) markers derived from the candidate gene were also validated by association analysis in Pop 2 and Pop 3.

### Fluorescent Real-Time Quantitative RT-PCR

Analysis of *TaTGW-7A* transcript levels was performed using 2 × probe qPCR Mix (with ROX) (SinoGene, Beijing, China). The primer sets for *TaTGW-7A* (FQ), *Actin* (Actin, control) and the probe sequences were shown in **Supplementary Table S6**. Each sample was analyzed in triplicate. The relative qualification 2^-ΔΔ^*^C^*^t^ method (Livak and Schmittgen, 2001) was used to calculate *TaTGW-7A* transcript levels with *Actin* as the endogenous control and Wangshuibai as a reference sample.

### IAA Purification and Quantification

In this study, IAA was quantified in immature seeds (1 g) at 20 and 30 DPA from six low- and six high-TGW varieties with three repeats. These seeds were frozen immediately in liquid nitrogen, homogenized in 80% methanol with a mortar and pestle, and then the mixture was stored at -20°C. To analyze IAA content, the mixture was stirred overnight at 4°C, and impurities were removed by centrifugation at 10,000 × *g* for 20 min. The supernatant was passed through a 0.22-μm millipore filter and then pre-equilibrated with 5 mL of 100% methanol and 70% methanol, respectively, using a C_18_ Waters Sep Pak cartridge (Waters, Milford, MA, USA) (Hu et al., 2016). The filtrate was collected and analyzed by high performance liquid chromatography (HPLC).

The HPLC was equipped with an Eclipse XDB-C_18_ column (250 mm × 4.6 mm, 5 μm, Agilent, Palo Alto, CA, USA). The mobile phase consisted of solution A (methanol) and solution B (acetic acid solution, pH 3.6). The elution profile was for 38 to 42.2% A in B (linear gradient) from 0 to 9 min, for 42.2 to 38% A in B (linear gradient) from 9 to 10 min, and for 38% A in B (linear gradient) from 10 to 25 min. The flow rate was 1 mL min^-1^ and the UV-absorbance detector was set to 250 nm. An external standard method was used to calculate the IAA concentration according to the manufacturer’s instructions^9^.

### Statistical Analysis

The significant difference of effects between the two alleles of *TaTGW-7A* on TGW was analyzed by ANOVA, and estimation of candidate gene effect on phenotypic variation was performed with GLM. These statistical analyses were all finished via SPSS 20 statistical software^10^.

## Results

### Analysis of BSA-SLAF-seq Data

In total, 132,530 SLAF tags were acquired, and 11,571 were polymorphic between two parents and between DNA bulks. Nine hundred and forty SLAF tags were significantly correlated with TGW by SNP_index analysis, and 938 were screened and positioned onto wheat chromosomes (**Supplementary Tables S7** and **S8**). In order to detect the hot regions carrying candidate target genes, these SLAF tags were also analyzed by association-region analysis. The results indicated that twenty-five polymorphic SLAF tags closely related to TGW were identified on six hot regions, and located on chromosomes 2A, 3A, 4B, 6A, 7A, and 7B, respectively, through BLAT analysis (**Table 1**). Particularly, two hot regions on 7A (six SLAF tags) and 7B (seven SLAF tags) carried more than half of TGW-associated SLAF tags (**Table 1**). Two full-length ORFs were also found in the two hot regions by alignment of SLAF tags sequences with wheat genome sequences. Therefore, the 13 SLAF tags on 7A and 7B were selected for subsequent analysis.

**Table 1:** Twenty-five polymorphic SLAFs in six hot-regions associated with TGW.

Chromosome ID	Start	End	Genetic distance/cM	Associated Marker number	Transcripts
2A	68.14	68.14	0	3	0
3A	60.18	60.18	0	3	0
4B	65.7	65.7	0	3	0
6A	60.96	60.96	0	3	0
7A1	93.26	93.26	0	3	1
7A2	98.92	98.92	0	3	0
7B1	161.06	163.78	2.72	3	0
7B2	168.73	170.36	1.63	4	1
Total				25	2

Of the 13 SLAF tags, SLAF65386 in the hot region of 7AS was 100% identical to the sequence 7AS_4248784 containing a full-length ORF, Traes_7AS_378A12AA9.1, through BLAST analysis in the Genetic and Genomic Information System Database^11^ (Supplementary Figures S1 and S2). The gene corresponding to the transcript Traes_7AS_378A12AA9.1 was designated as *TaTGW-7A*. Simultaneously, the sequence of SLAF65386 was extended 50 kb forward and reverses, and used for functional annotation and gene/transcript set/pathway enrichment analyses. The results indicated the transcript of *TaTGW-7A* (Traes_7AS_378A12AA9.1) could be annotated in the COG database, GO database, and KEGG database, respectively (**Supplementary Table S9**). The gene was predicted to encode a protein with a TIM barrel fold the same as IGPS. Therefore, *TaTGW-7A* could be regarded as a candidate gene for TGW on chromosome 7AS. In the present study, the putative gene associated with TGW on 7B was not obtained (data not shown).

### Genome-Wide QTL Location for TGW Using SSR Markers

In this study, several stable QTL for TGW were mapped on chromosomes 1B, 3A, 3D, 4B, 4D, 5A, 6A, 6B, 7A, and 7B (Supplementary Figure S3). These loci could explain 5.7–28.9% phenotypic variations of TGW in different environments (**Supplementary Table S10**). Moreover, the loci on 3A, 4B, 6A, 7A, and 7B could be detected both by SNP_index analysis and QTL mapping. Thereinto, the loci on 7A and 7B generally had higher LOD values than other loci in different environments. The result of QTL mapping was generally consistent with hot regions analysis for TGW through comparing the detected loci both by super-BSA and linkage analysis.

### Cloning and Characterization of *TaTGW-7A*

**FIGURE 1:**
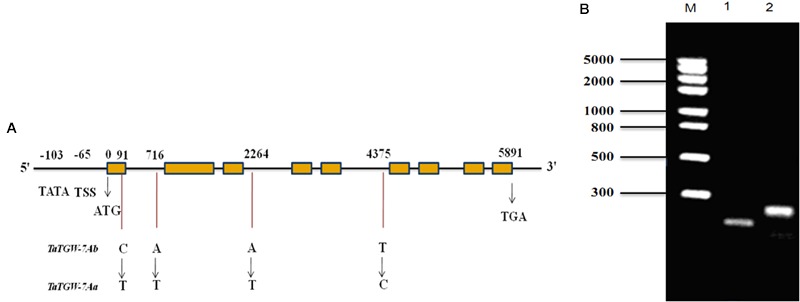
**(A)** Structure and allelic variations of *TaTGW-7A* gene. Four SNPs are present between *TaTGW-7Aa* and *TaTGW-7Ab*. The CAPS marker (TGW7A) is developed based on the SNP in the first exon (C-T). The TATA-box, transcription start site (TSS), start codon (ATG) and stop codon (TGA) are shown. **(B)** The CAPS marker is used for discriminating *TaTGW-7Aa* and *TaTGW-7Ab* on 2.0% agarose gel. M: marker; 1: Jing 411 (*TaTGW-7Aa*); 2: Hongmangchun 21 (*TaTGW-7Ab*).

The primer sets Q29, Q35, Q37, and SR were designed based on the sequences of 7AS_4248784 and Traes_7AS_378A12AA9.1 to amplify the full-length gDNA and cDNA sequences of *TaTGW-7A* from Jing 411 and Hongmangchun 21, respectively. The amplified fragments by primer sets 29P and 31P were used for assembling the full-length gDNA of *TaTGW-7A* (**Supplementary Table S6**). All amplified products were recovered, cloned, sequenced and assembled. Sequence analysis revealed that the Jing 411 allele had a full-length gDNA of 5891 bp, containing an ORF of 2241 bp with nine exons and eight introns (**Figure 1A**). The gene encodes a peptide of 746 amino acids with a predicted molecular weight of 37.7 kDa. Four nucleotide substitutions were present between Jing 411 and Hongmangchun 21 alleles (Supplementary Figure S4). Only one SNP in the first exon of *TaTGW-7A* was found consistently between the high- and low-TGW genotypes (**Supplementary Table S11**). This mutation resulted in a change from an alanine codon (GCC) in the low-TGW to a valine codon (GTC) in the high-TGW varieties. The TATA box was located at -103 position, and the transcription start site (TSS) was located at -65 position from the initiation codon (**Figure 1A**).

A signal peptide with a cleavage site located at positions 28–29 was predicted by the SignalP program (Supplementary Figure S5). Analysis of domain architectures in NCBI indicated that the functional domain of *TaTGW-7A* was an N-terminal domain of sigma 54-dependent transcriptional activators, the same as IGPS^12^ involved in the tryptophan biosynthetic pathway, which can catalyze the conversion of 1-[(2-carboxyphenyl) amino]-1-deoxyribulose 5-phosphate to indole-3-glycerol phosphate in IAA biosynthesis process (Supplementary Figures S6 and S7). The complete mRNA sequence of *TaTGW-7A* has been submitted to NCBI (KT582299).

### Development and Validation of *TaTGW-7A* Functional Marker

A CAPS marker (*TGW7A*) was developed based on the SNP in the first exon between two alleles of *TaTGW-7A*. A 250-bp fragment was amplified from both Jing 411 and Hongmangchun 21 using the primer set MQ (**Supplementary Table S6**). After digestion with *Bsm*AI at the SNP site (NEB, Nanjing, China), two fragments of 250 bp vs. 196 bp clearly distinguished between the Jing 411 and Hongmangchun 21 alleles, designated as *TaTGW-7Aa* and *TaTGW-7Ab*, respectively (**Figure 1B**). We further sequenced approximately 3 kb downstream of 7AS_4248784, and detected a 5-bp insertion-deletion (InDel) polymorphism between *TaTGW-7Aa* and *TaTGW-7Ab*. An InDel marker (*TG9*) was also developed, co-segregating with *TGW7A* in the RIL population. In addition, other four CAPS markers were designed based on polymorphic SLAFs on chromosome 7AS (**Supplementary Table S6**), designated as *SLAF49035, SLAF28300, SLAF133263*, and *SLAF6258*, respectively.

Three populations (Pop 1, Pop 2, and Pop 3) were used to validate the markers *TGW7A* and *TG9*, which were significantly associated with TGW in all environments, and the markers also showed significant association with GL and GW in Pop 1 and Pop 3 (**Table 2**).

**Table 2:** Association analysis of grain traits with *TaTGW-7A* genotypes in Pop 1 (RIL population), Pop 2 (natural population), and Pop 3 (Chinese mini-core collections).

Year	Grain traits^a^	*TaTGW-7Aa*	*TaTGW-7Ab*	*F*	PVE (%)
		(Mean ± SD)	(Mean ± SD)		
**Chinese wheat mini-core collections**					
2015	TGW(g)	34.14 ± 9.27	27.35 ± 6.99	36.92^∗∗^	12.3
	GL(mm)	6.50 ± 0.57	5.94 ± 0.46	64.62^∗∗^	19.9
	GW(mm)	3.11 ± 0.30	2.89 ± 0.28	32.31^∗∗^	10.9
**Natural population**					
2012	TGW(g)	38.77 ± 4.50	34.52 ± 7.17	21.94^∗∗^	8.9
2013	TGW(g)	42.15 ± 4.61	37.08 ± 7.65	29.12^∗∗^	12.4
2014	TGW(g)	44.20 ± 4.18	40.38 ± 6.84	20.22^∗∗^	7.6
**RIL population**					
2008	TGW(g)	41.24 ± 10.73	30.14 ± 5.98	52.56^∗∗^	26.2
2011	TGW(g)	38.16 ± 9.05	29.91 ± 4.95	41.00^∗∗^	21.7
2012	TGW(g)	38.83 ± 9.79	29.92 ± 4.14	43.77^∗∗^	22.8
2014	TGW(g)	38.42 ± 11.04	28.37 ± 4.38	44.26^∗∗^	23.0
2015	TGW(g)	34.71 ± 8.10	25.84 ± 5.40	54.88^∗∗^	27.1
	GL(mm)	6.51 ± 0.73	5.78 ± 0.30	51.86^∗∗^	25.9
	GW(mm)	3.18 ± 0.33	2.89 ± 0.15	40.93^∗∗^	21.7

*^a^TGW, thousand-grain weight; GL, grain length; GW, grain width; PVE, phenotypic variance explained by *TaTGW-7A*. Significant difference of TGW between the two alleles at the levels of 0.05 and 0.01 was marked with ^∗^ and ^∗∗^, based on *F*-test. The two markers, *TGW7A* and *TG9* have same results to discriminate alleles of TaTGW-7A.*

### Validation of *TaTGW-7A* by Linkage Analysis

**FIGURE 2:**
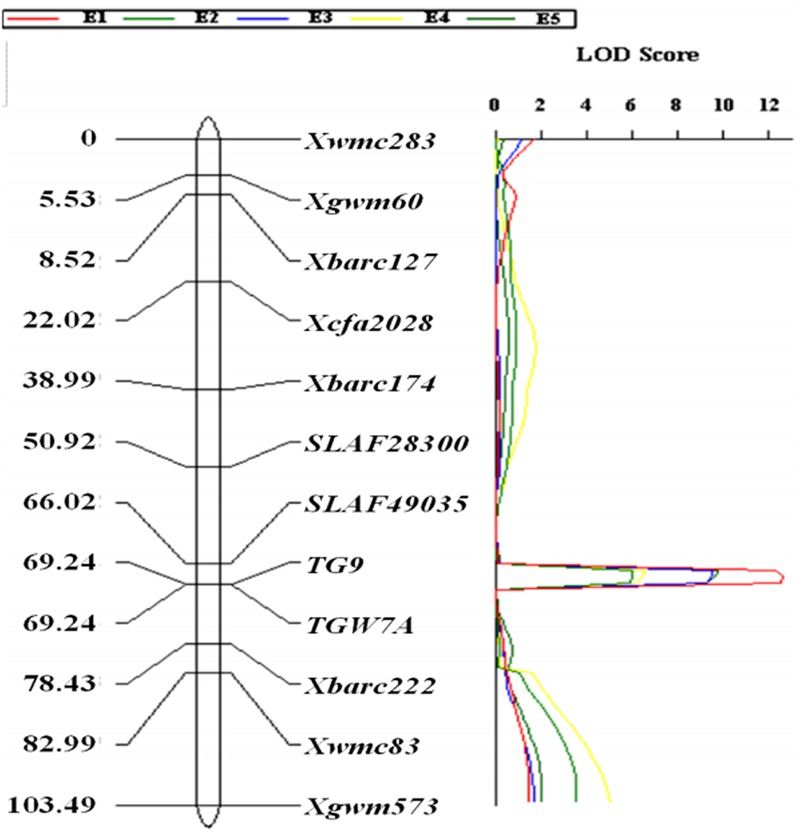
**Linkage mapping of *TaTGW-7A* on chromosome 7A in the RIL population derived from the cross of Jing 411 × Hongmangchun 21**.

The gene-specific markers *TGW7A* and *TG9*, two CAPS (*SLAF49035* and *SLAF28300*) as well as eight SSR markers were integrated into a linkage map for chromosome 7A, spanning a genetic distance of 103.49 cM (**Figure 2**). QTL analysis revealed that a stable QTL at *TaTGW-7A* locus was located in the interval of *SLAF49035* and *TGW7A/TG9* on chromosome 7AS, and explained 21.7–27.1% of the TGW variation in the RIL population across five environments (**Table 2**). In the genome-wide QTL mapping using SSR markers, a main locus was also detected between the *Xbarc174* and *Xbarc222*, which covered the interval of *SLAF49035* and *TGW7A/TG9* (**Figure 2**; Supplementary Figure S3). In addition, the *TaTGW-7A* locus was located on C-7AS-8 (0.45) according to the physical map of common wheat, as shown in Supplementary Figure S8 (Qi et al., 2004; Sourdille et al., 2004).

### *TaTGW-7A* Expression

**FIGURE 3:**
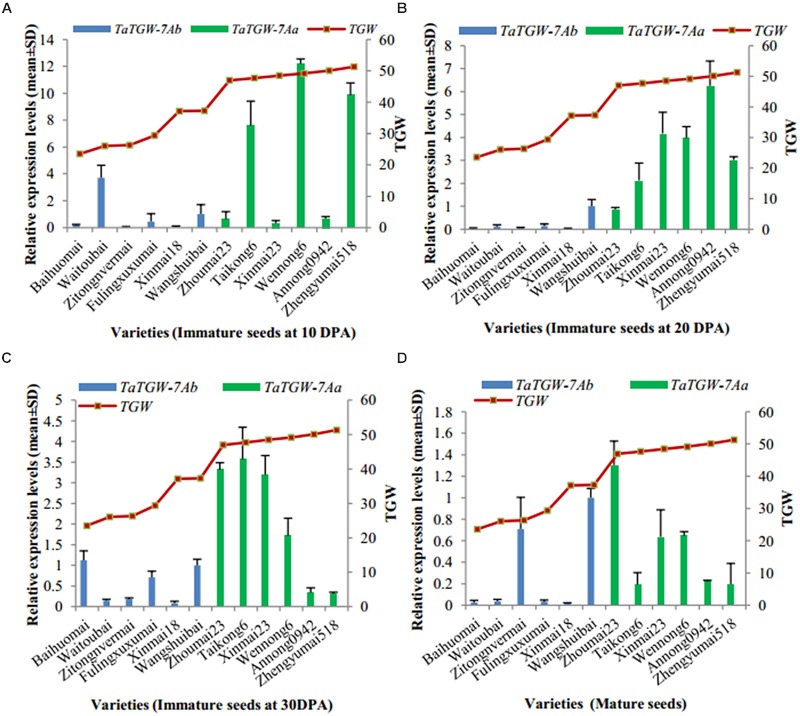
**Relationship between TGW and *TaTGW-7A* expression in immature and mature seeds. (A–D)** means the four stages from 10 DPA to mature.

Immature (10, 20, and 30 DPA) and mature seeds were used to analyze the expression level of *TaTGW-7A* (**Supplementary Table S6**). Generally, the relative transcript level of *TaTGW-7A* in immature seeds at 20 and 30 DPA was associated with TGW. The average transcript level of *TaTGW-7Aa* for high TGW was generally higher than those of *TaTGW-7Ab* for low TGW (**Figure 3**).

### Analysis of IAA Content

**FIGURE 4:**
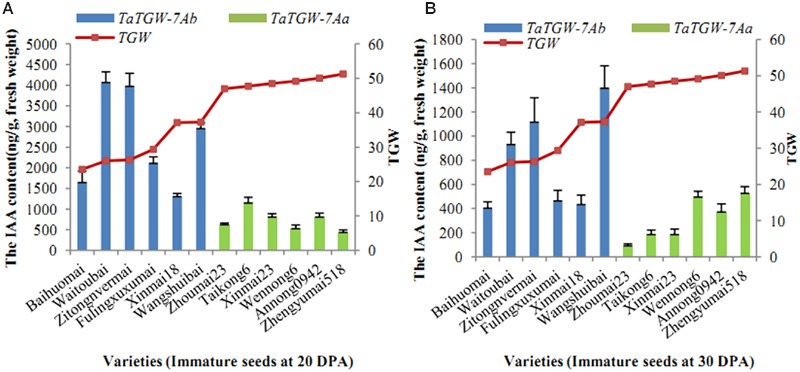
**Relationship between IAA content of *TaTGW-7A* genotype and TGW. (A,B)** means the two stages of 20 DPA and 30 DPA.

Six varieties with low and six with high TGW were sampled at 20, 30 DPA and IAA contents were determined. The results showed that IAA content was related to TGW. The average level of IAA in wheat varieties with *TaTGW-7Ab* was generally higher than those in *TaTGW-7Aa* genotypes at 20 and 30 DPA (**Figure 4**).

### Frequency Distributions of *TaTGW-7A* Alleles

A high frequency of *TaTGW-7Aa* was found in Chinese mini-core collections (Pop3, 59%), and in most of Chinese wheat production areas, including the Low and Middle Yangtze River Valley winter wheat region (62%), Southern winter wheat region (67%), Northeastern spring wheat region (100%), Northern spring wheat region (89%), Northwestern spring wheat region (62%), Qinghai-Tibet spring-winter wheat region (86%), and Xinjiang winter-spring wheat region (100%) (Supplementary Figure S9; **Supplementary Tables S12** and **S13**). In contrast, the *TaTGW-7Ab* allele showed higher frequencies in Northern winter wheat production area (61%), and Southern winter wheat production area (51%). In Pop 4, *TaTGW-7Aa* was a predominant allele distributed in most of wheat areas, including Northern winter wheat region (89%), Yellow and Huai River valley winter wheat region (90%), Low and middle Yangtze River valley winter wheat region (88%), Southwestern winter wheat region (74%), Northeastern spring wheat region (77%), Northwestern spring wheat region (81%) and Xinjiang winter-spring wheat region (67%) (**Supplementary Table S13**).

## Discussion

### Application of Super-BSA and Bio-information to Isolate Genes in Common Wheat

Wheat is an allohexaploid species with extremely large genomes and a detailed physical map has not been available for most of wheat chromosomes. Moreover, wheat genome contains a high proportion (>80%) of repeated sequences (Eilam et al., 2007; Wicker et al., 2011), making map-based cloning difficult and time consuming. The SLAF-seq for marker development is highly efficient, inexpensive, and applicable for analyses of large populations; hence, it has been proposed as an effective method for marker-assisted breeding (Sun et al., 2013). SLAF-seq in combination with BSA is a simple and efficient approach to detect candidate genes and develop gene-specific markers (Trick et al., 2012; Chen et al., 2013; Qin et al., 2015; Xu et al., 2015c). However, this approach has not been frequently used for marker development and isolation of functional genes in common wheat.

In this study, we identified one candidate gene *TaTGW-7A* associated with TGW on the hot region of 7AS through super-BSA analysis. In addition, eight genes were predicted by functional annotation and gene/transcript set/pathway enrichment analyses of six SLAF tags on the hot region of 7A (**Supplementary Table S9**); *TaTGW-7A* was more significantly associated with TGW by linkage analysis, compared with the other SLAF tags on 7A (**Figure 2**). Moreover, the mapping analysis of *TaTGW-7A* was generally consistent with raw QTL mapping using SSR markers (**Figure 2**; Supplementary Figure S3). Thus, it is worthy of further focusing the SLAF65386 in the hot region of 7AS, corresponding to Traes_7AS_378A12AA9.1 with full-length ORF. In addition, identification of *TaTGW-7A* can be considered as an effective and accurate application of super-BSA in development of molecular markers and isolation of candidate genes from wheat. However, for polyploid plants such as common wheat, it is very difficult to detect minor genes by BSA-SLAF-seq, and SLAF-seq cannot cover the whole genome for gene discovery. For example, several QTL for TGW were detected on 1B, 3A, 3D, 4B, 4D, 5A, 6A, 6B, 7A, and 7B, respectively; however, we only identified a candidate gene, *TaTGW-7A*, on 7A by super-BSA and bio-information analysis. Another hot region associated with TGW on 7B probably comprised candidate genes; however, we could not identify them via super-BSA. This is possibly because these SLAF tags do not exist in or have a long distance from the candidate gene and no enough information of genome sequence for analysis in common wheat, compared with rice. In addition, SLAF tags on 2A, 3A, 4B, and 6A also showed close association with TGW, which are also worthy of further developing CAPS markers. Therefore, other methods should also be used for identifying functional genes in wheat. In our previous studies, several genes including *TaCKOX6a02* on 3DS (Lu et al., 2015), *Tackx4* on 3AL (Chang et al., 2015), and *TaTGW6* on 4AL (Hu et al., 2016) associated with TGW were identified based on the homology analysis of rice genes.

### Characterization and Putative Molecular Mechanism of *TaTGW-7A*

The *TaTGW-7A* gene contained nine exons and eight introns. Four SNPs were detected between two alleles, *TaTGW-7Aa* and *TaTGW-7Ab*, three of which were in introns and one in exon. The SNP in the first exon of *TaTGW-7A* resulted in an amino acid change from Val to Ala at residue 31, corresponding to a change from a high-TGW to a low-TGW phenotype. This suggested the point mutation possibly affected protein stability because of changes in the secondary structure^13^. Two alpha helixes and one beta sheet occurred at the amino acid mutation site of *TaTGW-7Ab*, compared with *TaTGW-7Aa*. The changes in secondary structure possibly influence the function of *TaTGW-7A* (Cheng et al., 2006). The deduced amino acid sequences showed the presence of highly conserved TIM-br_sig_trns and UPF0261 functional domains, forming a TIM barrel fold structure, the same as IGPS^14^, which was a key enzyme involved in tryptophan synthesis and ultimately affected IAA biosynthesis. The domain was found as an N-terminal domain of sigma 54-dependent transcriptional activators (enhancer-binding proteins), suggesting a potential role in signal recognition/reception and signal transduction (Bush and Dixon, 2012).

To explore the potential molecular mechanism of *TaTGW-7A*, we analyzed transcript expression of *TaTGW-7A* and corresponding IAA contents in wheat varieties. The transcript level of *TaTGW-7A* was significantly correlated with TGW in immature seeds at 20 and 30 DPA, and the IAA content was negatively correlated with TGW. Compared with *TaTGW-7Ab* genotype, *TaTGW-7Aa* corresponded to higher TGW and lower IAA content. It was assumed that *TaTGW-7A* was required for synthetic regulation of IAA. The higher expression of *TaTGW-7Aa* possibly reduced the biosynthesis of tryptophan and IAA.

Ishimaru et al. (2013) reported that *TGW6*, encoding an IAA-glucose hydrolase, could increase GL and TGW in the Indian rice landrace Kasalath. The *tgw6* allele in Nipponbare affected the timing of the transition from syncytial to cellular phases by controlling IAA supply through IAA-glucose hydrolase activity, and consequently limited cell number and GL. In our previous study, *TaTGW6* gene on chromosome 4AL was cloned using a comparative genetics approach (Hu et al., 2016). Our results revealed that low IAA contents in immature seeds at middle (20 DPA) and late grain filling stages (30 DPA) were significantly associated with low transcript levels of *TaTGW6*, corresponding to high TGW. Our previous study showed that the IAA content in immature seeds at the middle and late stages of grain filling (20–30 DPA) was significantly related to grain weight (Hu et al., 2016). Taken together, these results suggest that grain weight is negatively regulated by IAA at the middle to late stages of grain filling. Different from *TGW6* of rice and *TaTGW6* of wheat, *TaTGW-7A* positively regulates the grain weight of wheat. It is noteworthy that *TaTGW-7A, TGW6*, and *TaTGW6* are all associated with grain weight and involved in IAA biosynthesis and metabolism. These findings suggest that IAA level has a close relationship with grain development. Nevertheless, elucidation of the molecular mechanism underlying *TaTGW-7A* regulation of grain development will require more detailed research.

### QTL for Grain Weight on Chromosome 7A

In previous studies, QTL for grain weight have been detected on almost all 21 wheat chromosomes in different segregating populations (Shah et al., 1999; Kato et al., 2000; Ammiraju et al., 2001; Börner et al., 2002; Groos et al., 2003; Huang et al., 2003, 2006; Quarrie et al., 2005; Breseghello and Sorrells, 2006; Kumar et al., 2006; Li et al., 2007; Liao et al., 2008; Sun et al., 2009; Lopes et al., 2013; Simmonds et al., 2014). Several QTL for TGW or grain size on chromosome 7A have been reported (Huang et al., 2003; Kumar et al., 2006, 2007; Kuchel et al., 2007; Su et al., 2016). However, no TGW gene on chromosome 7A has been cloned so far. In the present study, a major QTL at *TaTGW-7A* locus for TGW, GL and GW, was also detected on chromosome 7AS linked to *Xbarc222*, and it was located to chromosome C-7AS-8 (0.45) based on the reports of Qi et al. (2004) and Sourdille et al. (2004). This location differs from those of the previously reported QTL clusters (Kumar et al., 2007). Therefore, *TaTGW-7A* is likely to be a novel gene associated with TGW in wheat.

### Strong Positive Selection of *TaTGW-7Aa* in Wheat Breeding

As an important agronomical trait, high TGW is always the target of selection in crop improvement; therefore, underlying genes involved in TGW variation have been selected during the long-term breeding process. For example, *TaGW2-6A* (*Hap-6A-A*) associated with TGW has undergone positive selection in wheat breeding (Su et al., 2011). Similarly, *TaGS-D1* associated with TGW and GL showed a high frequency of distribution in Chinese wheat varieties and underwent strong positive selection during the breeding process (Zhang et al., 2014). *TaGS5-3A*, a gene underlying grain size, also underwent strong artificial selection during wheat polyploidization events (Ma et al., 2016).

In this study, the superior allele *TaTGW-7Aa* showed a significantly higher frequency than *TaTGW-7Ab* both in Chinese mini-core collections and 501 wheat varieties, especially in modern wheat varieties. In addition, *TaTGW-7Aa* was found to be widely distributed among 10 ecological wheat production zones of China, indicating that this allele has been strongly selected in Chinese wheat breeding. The *TGW7A* and *TG9* markers developed in this study can be used directly for TGW improvement in wheat breeding. In particular, the InDel marker *TG9* segregating with *TaTGW-7A* is more easily used for screening desirable lines for the multi-gene pyramiding in wheat breeding.

## Conclusion

*TaTGW-7A*, an IGPS-like gene, was cloned and characterized via super-BSA and bio-information analysis. Two alleles, *TaTGW-7Aa* and *TaTGW-7Ab*, could be discriminated by CAPS marker *TGW7A* and InDel marker *TG9* designed based on the sequences of *TaTGW-7A*. The gene was located on chromosome 7AS and 7.6–27.1% of phenotypic variations of TGW could be explained by the locus in different environments and genetic backgrounds. *TaTGW-7Aa* showed higher transcript levels and corresponded to significantly higher TGW but lower IAA content during grain development, compared with *TaTGW-7Ab*. *TaTGW-7Aa* has been strongly and positively selected in wheat breeding in China. The molecular markers *TGW7A* and *TG9* can be used for improvement of TGW in breeding programs.

## Ethical Standards

The experiments conducted in this study comply with the current laws of China.

## Author Contributions

CC and C-XM initiated the project and designed the experiment; M-JH, H-PZ, and CC conducted gene cloning, marker development, mapping analysis, and prepared the manuscript; M-JH, XZ, Z-YW, HJ, J-JC, JL, and S-XW performed all tests of grain size, weight and IAA content; X-CX and G-LS assisted in writing the paper; all authors read and approved the final manuscript.

## Conflict of Interest Statement

The authors declare that the research was conducted in the absence of any commercial or financial relationships that could be construed as a potential conflict of interest.

## Footnotes

1 http://www.biomarker.com.cn/

2 https://www.ncbi.nlm.nih.gov

3 http://wheat-urgi.versailles.inra.fr/

4 www.cerealsdb.uk.net

5 http://www.lynnon.com

6 http://www.softberry.com

7 http://wheat.pw.usda.gov

8 http://www.isbreeding.net

9 http://www.agilent.com/home

10 http://www-01.ibm.com/software/analytics/spss/

11 http://wheat-urgi.versailles.inra.fr/

12 http://www.ncbi.nlm.nih.gov/

13 http://linux1.softberry.com/berry.phtml

14 http://www.ncbi.nlm.nih.gov/

## Supplementary Material

TABLE S1 **Grain traits of the two parents, RIL population (Pop 1)**.

TABLE S2 **Grain weight and allelic variants in Pop 2**.

TABLE S3 **Grain traits and allelic variations of 257 Chinese wheat mini-core collections (Pop 3)**.

TABLE S4 **Allelic variation and geographic distribution for 501 accessions (Pop 4)**.

TABLE S5 **All polymorphic SLAF with detal_index**.

TABLE S6 **Primers for quantitative RT-PCR, functional markers and amplification of the full-length gDNA and cDNA**.

TABLE S7 **Significant association SLAF marker**.

TABLE S8 **Significantly associated SLAF tags on genome of common wheat**.

TABLE S9 **Genes predicted to associate with grain development**.

TABLE S10 **QTLs for TGW across five environments**.

TABLE S11 **Grain weight and allele variations of twelve varieties**.

TABLE S12 **The proportions of two *TaTGW-7A* alleles in RIL (Pop 1), natural population (Pop 2), and Chinese wheat mini-core collections (Pop 3)**.

TABLE S13 **Geographic distribution of *TaTGW-7A* alleles in Pop 3 and Pop 4**.

## Abbreviations

ANOVA: analyses of variance
CAPS: cleaved amplified polymorphic sequence
DPA: days post anthesis
GL: grain length
GLM: general linear model
GW: grain width
IGPS: indole-3-glycerol-phosphate synthase
ORF: open reading frame
QTL: quantitative trait locus/loci
RIL: recombinant inbred lines
RT-PCR: real-time quantitative PCR
SLAF-seq: specific-locus amplified fragment sequencing
SNP: single nucleotide polymorphism
SSR: simple sequence repeat
Super-BSA: combination of SLAF-seq and BSA analysis
TGW: thousand-grain weight
