# Supplementary material for: Cloning and Characterization of TaTGW-7A Gene Associated with Grain Weight in Wheat via SLAF-seq-BSA
Source: Front Plant Sci. 2016 Dec 20;7:1902. doi: 10.3389/fpls.2016.01902 (PMC5167734; doi:10.3389/fpls.2016.01902)

Supplementary materials

Cloning and characterization of *TaTGW-7A* gene associated with grain weight in wheat via specific-locus amplified fragment sequencing and bulked segregant analysis

Ming-Jian Hu[1†], Hai-Ping Zhang[1†], Kai Liu[1], Jia-Jia Cao[1], Sheng-Xing Wang[1], Hao Jiang[1], Zeng-Yun Wu[1], Jie Lu[1], Xiao Feng Zhu[1], Xian-Chun Xia [1,2], Gen-Lou Sun [1,3], Chuan-Xi Ma[1] , Cheng Chang *[1]

[1] Key Laboratory of Wheat Biology and Genetic Improvement on Southern Yellow & Huai River Valley, the Ministry of Agriculture/College of Agronomy, Anhui Agricultural University; Hefei, China

[2] National Wheat Improvement Center/The National Key Facility for Crop Gene Resources and Genetic Improvement, Institute of Crop Science, Chinese Academy of Agricultural Sciences (CAAS), Beijing, China

[3]Department of Biology, Saint Mary's University, Halifax, NS B3H3C3, Canada

* Correspondence: Cheng Chang (changtgw@126.com)

1. Supplementary Tables
2. Supplementary Figures

1. Supplementary Tables

Table S1 Grain traits of the two parents, RIL population (Pop 1)

Traits[a]	Parents		RIL population (n =150)	
	Jing411	Hongmangchun 31	Mean	C.V.%
GL (mm)	6.87	5.41	6.37(4.62-7.31)	25.33
GW (mm)	3.83	2.91	3.08(2.34-4.07)	17.13
TGW(g)	47.6	19.7	38.1(23.4-56.2)	31.04

[a]The means performance of grain traits were measured in five cropping seasons (RIL population). The data in parentheses mean the range of phenotypic values. GL, grain length; GW, grain width; TGW, 1000-grain-weight; RIL, recombinant inbred line.

Table S2 Grain weight and allelic variants in Pop 2

Accession	Grain weight (g)			*TaTGW-7A* allele
	2012	2013	2014	
984121	37.24	42.05	41.97	*TaTGW-7Aa*
02P67	37.61	42.11	45.30	*TaTGW-7Aa*
02Y151	43.52	45.77	48.27	*TaTGW-7Aa*
03-885	34.57	37.95	41.97	*TaTGW-7Aa*
03G7	37.11	38.99	52.18	*TaTGW-7Aa*
Aifengzao8	40.55	44.66	44.82	*TaTGW-7Aa*
Aikang58	38.67	41.36	40.37	*TaTGW-7Aa*
Aizao64	38.33	41.10	43.80	*TaTGW-7Aa*
Annong0807	39.32	42.23	42.11	*TaTGW-7Aa*
Annong0932	43.45	46.65	47.03	*TaTGW-7Aa*
Annong0942	37.23	43.87	42.52	*TaTGW-7Aa*
Annong0942-13	45.26	50.50	54.51	*TaTGW-7Aa*
Annong1001	44.63	47.68	42.54	*TaTGW-7Aa*
Annong1014	40.69	38.35	45.72	*TaTGW-7Aa*
Annong1039	42.30	42.23	46.70	*TaTGW-7Aa*
Annong1106	40.80	45.42	44.23	*TaTGW-7Aa*
Annong1107	39.82	41.00	45.78	*TaTGW-7Aa*
Annong1108	42.05	48.55	48.58	*TaTGW-7Aa*
Annong1110	41.10	43.85	47.58	*TaTGW-7Aa*
Annong1114	41.25	42.62	47.21	*TaTGW-7Aa*
Annong8455	37.69	41.89	47.39	*TaTGW-7Aa*
Annong92484W	34.34	31.20	46.50	*TaTGW-7Aa*
Annong9267	29.35	34.86	37.02	*TaTGW-7Aa*
AR2	31.99	33.29	35.27	*TaTGW-7Aa*
Baimanghong	26.35	28.36	24.72	*TaTGW-7Aa*
Bainong207	45.58	43.70	42.27	*TaTGW-7Aa*
Bainong3271	36.81	38.10	41.73	*TaTGW-7Aa*
Bainong64	36.12	36.89	41.38	*TaTGW-7Aa*
Bainongaikang58	40.98	41.80	42.04	*TaTGW-7Aa*
Baipi224	42.53	47.22	51.82	*TaTGW-7Aa*
Baitumai	29.47	30.50	33.94	*TaTGW-7Aa*
Baiyuhua	34.28	38.90	39.89	*TaTGW-7Aa*
Baofeng10-82	37.95	48.60	47.78	*TaTGW-7Aa*
Baomai3	35.69	46.41	47.04	*TaTGW-7Aa*
Baomai8	32.79	40.53	39.72	*TaTGW-7Aa*
Bolunxuan182	43.32	49.50	46.39	*TaTGW-7Aa*
Cang97-051	30.06	33.47	37.86	*TaTGW-7Aa*
Cangmai119	32.16	40.09	42.79	*TaTGW-7Aa*
Chuanmai42	45.51	50.32	50.57	*TaTGW-7Aa*
CP02-63-13-1	38.70	41.57	39.96	*TaTGW-7Aa*

CP02-8-5-6-1	40.61	40.59	40.20	*TaTGW-7Aa*
CP02-9-3-1-1-1	44.92	46.03	48.16	*TaTGW-7Aa*
CP20-39-11-1	36.26	35.89	42.08	*TaTGW-7Aa*
Dangmai2	37.13	39.70	45.99	*TaTGW-7Aa*
Danshi802	39.23	45.67	45.34	*TaTGW-7Aa*
Dinghong208	42.28	50.24	50.80	*TaTGW-7Aa*
ENESCO	34.13	36.12	35.16	*TaTGW-7Aa*
Fanmai5	40.97	42.64	51.46	*TaTGW-7Aa*
Fanmai8	44.76	41.09	42.43	*TaTGW-7Aa*
FARO	32.34	36.28	41.51	*TaTGW-7Aa*
Fu0382	39.98	50.57	42.73	*TaTGW-7Aa*
Gaocheng8901	36.75	35.22	40.09	*TaTGW-7Aa*
Gaomai119	29.91	31.78	41.68	*TaTGW-7Aa*
Gaoyou9415	33.46	35.96	40.07	*TaTGW-7Aa*
Gaoyou9618	32.33	36.99	38.56	*TaTGW-7Aa*
Glenlen	36.66	41.28	46.59	*TaTGW-7Aa*
Gouyou9409	35.74	39.78	43.37	*TaTGW-7Aa*
Guan35	38.25	35.70	41.49	*TaTGW-7Aa*
Guinong775	40.63	40.05	45.73	*TaTGW-7Aa*
Guomai0608	41.83	43.22	49.20	*TaTGW-7Aa*
Guomai10	35.91	47.58	46.01	*TaTGW-7Aa*
Guomai8	42.59	44.75	50.94	*TaTGW-7Aa*
Guoshengmai1	36.08	39.99	39.25	*TaTGW-7Aa*
Han4564	34.33	39.97	48.04	*TaTGW-7Aa*
Han5030	41.73	40.13	40.21	*TaTGW-7Aa*
Han9565	33.11	34.17	41.24	*TaTGW-7Aa*
Heng4338	36.18	40.50	40.33	*TaTGW-7Aa*
Heng6149	32.48	41.31	46.92	*TaTGW-7Aa*
Heng7228	27.49	39.45	46.36	*TaTGW-7Aa*
Hengguan35	41.04	40.63	41.16	*TaTGW-7Aa*
Hengyou18	32.89	42.29	45.91	*TaTGW-7Aa*
Henong326	43.53	44.04	50.97	*TaTGW-7Aa*
Henong638	38.55	45.62	46.64	*TaTGW-7Aa*
Henong825	38.57	43.39	45.07	*TaTGW-7Aa*
Henong972	39.78	44.13	41.31	*TaTGW-7Aa*
Henongkangbai4	33.29	42.54	37.70	*TaTGW-7Aa*
Hongwan3	39.26	43.85	47.57	*TaTGW-7Aa*
Huaimai0320	37.91	37.55	43.86	*TaTGW-7Aa*
Huaimai0882	40.92	46.01	46.70	*TaTGW-7Aa*
Huaishi0806	39.16	50.61	48.99	*TaTGW-7Aa*
Huapei0616H-119	36.99	40.74	43.37	*TaTGW-7Aa*
Huarui0712	42.77	43.61	46.74	*TaTGW-7Aa*
Hulutou	33.47	34.83	34.99	*TaTGW-7Aa*
Jimai035037	38.36	41.13	41.91	*TaTGW-7Aa*

Jimai056487	44.26	54.72	44.35	*TaTGW-7Aa*
Jimai19	37.59	46.82	45.19	*TaTGW-7Aa*
Jimai20	38.52	39.97	43.74	*TaTGW-7Aa*
Jimai5319	41.36	48.38	46.30	*TaTGW-7Aa*
Jimai7251--1	36.25	37.10	41.62	*TaTGW-7Aa*
Jimai7251--2	35.01	47.62	46.84	*TaTGW-7Aa*
Jimai73	34.00	36.89	45.82	*TaTGW-7Aa*
Jinfeng0459	40.79	36.28	38.66	*TaTGW-7Aa*
Jinfeng6164	37.25	41.97	45.01	*TaTGW-7Aa*
Jining16	43.26	49.57	47.16	*TaTGW-7Aa*
Jinli88	36.17	44.66	43.00	*TaTGW-7Aa*
Jinmai31	44.40	39.19	41.91	*TaTGW-7Aa*
Jinnong4	39.50	40.83	42.96	*TaTGW-7Aa*
Junmai35	39.55	47.84	49.30	*TaTGW-7Aa*
Lankao298	41.24	42.68	42.03	*TaTGW-7Aa*
Lemai091156	42.90	45.67	39.71	*TaTGW-7Aa*
Linmai2	42.75	37.76	51.25	*TaTGW-7Aa*
Longke0901	38.65	43.17	44.94	*TaTGW-7Aa*
Lumai23	40.93	35.78	48.17	*TaTGW-7Aa*
Luo6099	39.58	39.43	42.46	*TaTGW-7Aa*
Luo9920	38.63	45.81	44.27	*TaTGW-7Aa*
Luomai21--1	38.44	43.58	46.60	*TaTGW-7Aa*
Luomai21--2	42.79	47.36	45.54	*TaTGW-7Aa*
Luyuan502	43.84	47.86	50.03	*TaTGW-7Aa*
M010	37.91	42.49	37.42	*TaTGW-7Aa*
M013	50.20	52.88	50.71	*TaTGW-7Aa*
M015	39.01	44.60	43.47	*TaTGW-7Aa*
M019	39.90	43.19	45.95	*TaTGW-7Aa*
M040	44.05	45.68	45.80	*TaTGW-7Aa*
M046	46.29	49.35	49.30	*TaTGW-7Aa*
M051	40.68	43.98	49.43	*TaTGW-7Aa*
M094	41.08	43.81	44.15	*TaTGW-7Aa*
M108	45.39	49.90	51.12	*TaTGW-7Aa*
M126	38.67	46.35	45.57	*TaTGW-7Aa*
Mingtian0417	40.33	41.53	45.06	*TaTGW-7Aa*
Neimai10	36.40	42.86	47.11	*TaTGW-7Aa*
Neixiang203	36.87	40.94	44.43	*TaTGW-7Aa*
Niavt14	33.97	37.08	37.62	*TaTGW-7Aa*
Qian079984-14	24.67	42.17	38.30	*TaTGW-7Aa*
Qianmai18	33.26	43.41	41.81	*TaTGW-7Aa*
R146	38.90	40.47	43.90	*TaTGW-7Aa*
S038186	37.89	43.47	46.74	*TaTGW-7Aa*
Shannong055843	37.40	40.04	41.02	*TaTGW-7Aa*
Shannong15	42.98	43.75	45.10	*TaTGW-7Aa*

Shigao02-1	39.10	36.48	38.26	*TaTGW-7Aa*
Shijiazhuang8	36.12	47.28	45.85	*TaTGW-7Aa*
Shimai12	42.76	45.27	49.01	*TaTGW-7Aa*
Shimai14	38.78	38.77	45.33	*TaTGW-7Aa*
Shimai15	38.46	40.89	40.42	*TaTGW-7Aa*
shimai16	36.65	37.73	41.56	*TaTGW-7Aa*
Shixin618	40.80	41.06	41.14	*TaTGW-7Aa*
Shixin703	40.63	41.21	41.89	*TaTGW-7Aa*
Shixin733	39.74	34.28	43.30	*TaTGW-7Aa*
Shiyou17	43.35	41.23	46.94	*TaTGW-7Aa*
Su553	44.84	46.97	45.41	*TaTGW-7Aa*
Su853	41.14	41.14	44.06	*TaTGW-7Aa*
Sumai3	37.08	36.20	40.53	*TaTGW-7Aa*
Tai10604	43.33	42.74	47.78	*TaTGW-7Aa*
Tai18	40.16	44.98	39.29	*TaTGW-7Aa*
Taikong6	46.97	47.60	48.55	*TaTGW-7Aa*
Taishan23	41.90	36.66	48.23	*TaTGW-7Aa*
Tuhulutou	33.44	37.76	39.54	*TaTGW-7Aa*
Wanke06290	36.29	44.32	38.54	*TaTGW-7Aa*
Wanke08585	42.14	41.67	46.43	*TaTGW-7Aa*
Wanke09636	44.87	42.69	50.86	*TaTGW-7Aa*
Wanmai38	36.15	41.28	38.38	*TaTGW-7Aa*
Wannong606	32.90	41.92	40.43	*TaTGW-7Aa*
Weierte1	39.55	40.65	44.83	*TaTGW-7Aa*
Wennong6	44.05	49.84	53.62	*TaTGW-7Aa*
X9610	36.24	35.22	41.09	*TaTGW-7Aa*
Xiannong1	38.21	49.73	46.61	*TaTGW-7Aa*
Xiaoyan6	36.02	39.10	40.90	*TaTGW-7Aa*
Xing05-4241	28.14	43.07	46.64	*TaTGW-7Aa*
Xingmai13	33.75	43.47	42.46	*TaTGW-7Aa*
Xingmai6	34.35	41.27	45.12	*TaTGW-7Aa*
Xinmai0401	35.10	47.45	49.88	*TaTGW-7Aa*
Xinmai19023	36.62	44.50	44.14	*TaTGW-7Aa*
Xinmai23	46.34	51.01	48.17	*TaTGW-7Aa*
Xinong622	39.86	44.04	42.72	*TaTGW-7Aa*
Xinong889	38.22	33.19	44.27	*TaTGW-7Aa*
Xinong979	31.43	44.10	47.44	*TaTGW-7Aa*
Xinyuanmai04130	42.32	45.06	42.92	*TaTGW-7Aa*
Xu5034	35.02	38.80	38.82	*TaTGW-7Aa*
Xuke1	43.90	44.84	49.72	*TaTGW-7Aa*
Xumai9074	40.79	49.37	45.49	*TaTGW-7Aa*
Y14	35.01	41.17	40.62	*TaTGW-7Aa*
Y18	33.48	38.68	39.65	*TaTGW-7Aa*
Yan2415	59.14	37.98	42.05	*TaTGW-7Aa*

Yangmai16	39.65	45.37	41.46	*TaTGW-7Aa*
Yangmai19	32.34	40.51	40.97	*TaTGW-7Aa*
Yangmai20	37.66	44.93	43.90	*TaTGW-7Aa*
Yangnuomai1	32.70	37.62	36.37	*TaTGW-7Aa*
Yannong19	45.73	44.63	42.42	*TaTGW-7Aa*
Yannong24	37.72	40.02	45.86	*TaTGW-7Aa*
Yannong24	36.21	36.55	43.14	*TaTGW-7Aa*
Yi5265	42.20	40.75	44.43	*TaTGW-7Aa*
Yi5385	42.08	40.56	43.34	*TaTGW-7Aa*
Yi95-5219	40.44	45.57	50.39	*TaTGW-7Aa*
Yi95-6023	37.51	37.15	44.59	*TaTGW-7Aa*
Yimai34	36.37	30.85	38.34	*TaTGW-7Aa*
Yishi02-1	36.99	41.60	45.46	*TaTGW-7Aa*
Yu0926	37.10	42.70	40.10	*TaTGW-7Aa*
Yumai2	41.05	37.87	46.30	*TaTGW-7Aa*
Yumai7	34.17	45.90	45.39	*TaTGW-7Aa*
Yunong69	43.44	42.48	39.12	*TaTGW-7Aa*
Zheng9023	39.82	44.54	51.79	*TaTGW-7Aa*
Zhengmai98	38.98	45.66	44.12	*TaTGW-7Aa*
Zhengnong19	39.72	42.51	39.43	*TaTGW-7Aa*
Zhengyumai518	48.53	49.56	55.80	*TaTGW-7Aa*
Zhongmai1187	45.46	48.14	49.39	*TaTGW-7Aa*
Zhongmai155	38.76	42.39	42.12	*TaTGW-7Aa*
Zhongmai895	43.48	45.83	46.75	*TaTGW-7Aa*
Zhongyou989	41.01	42.52	40.92	*TaTGW-7Aa*
Zhongyu1095	41.30	45.84	47.40	*TaTGW-7Aa*
Zhoumai11	39.60	39.37	41.34	*TaTGW-7Aa*
Zhoumai16	35.65	38.28	48.29	*TaTGW-7Aa*
Zhoumai17	30.11	38.92	42.26	*TaTGW-7Aa*
Zhoumai18	42.01	47.31	48.89	*TaTGW-7Aa*
Zhoumai19	37.18	40.10	42.37	*TaTGW-7Aa*
Zhoumai20	38.71	42.96	46.25	*TaTGW-7Aa*
Zhoumai22	41.59	43.38	45.65	*TaTGW-7Aa*
Zhoumai23	44.58	48.53	47.84	*TaTGW-7Aa*
Zhoumai25	34.04	31.72	42.68	*TaTGW-7Aa*
Zhoumai31	40.47	45.79	45.51	*TaTGW-7Aa*
Zi0706	37.19	40.61	42.83	*TaTGW-7Aa*
Annong1116	43.02	42.75	45.12	*TaTGW-7Ab*
Baihuomai	23.65	20.66	26.28	*TaTGW-7Ab*
Chadianhongmai	26.15	22.18	31.48	*TaTGW-7Ab*
E158	35.35	38.17	49.93	*TaTGW-7Ab*
Fengdecunmai5	43.73	46.46	47.67	*TaTGW-7Ab*
Gaoyou1817	35.60	32.35	36.34	*TaTGW-7Ab*
Heng87-6476	38.14	42.23	50.90	*TaTGW-7Ab*

Heng97-4119	34.55	43.31	43.43	*TaTGW-7Ab*
Huaimai0360	39.12	44.46	41.45	*TaTGW-7Ab*
Huangguaxian	28.24	27.20	31.41	*TaTGW-7Ab*
Huapei8	43.52	48.95	47.49	*TaTGW-7Ab*
Jingdong10	29.26	42.53	50.92	*TaTGW-7Ab*
Jinhe0459	37.22	33.84	42.97	*TaTGW-7Ab*
Langzhongbaimaizi	24.12	22.21	36.58	*TaTGW-7Ab*
Luo2267	34.08	37.43	41.01	*TaTGW-7Ab*
M104	42.45	45.65	45.56	*TaTGW-7Ab*
Mianmai37	30.58	42.23	41.35	*TaTGW-7Ab*
Neimai11	37.42	43.85	44.78	*TaTGW-7Ab*
Neimai8	42.93	45.40	41.96	*TaTGW-7Ab*
Peilingxuxumai	24.90	30.54	32.69	*TaTGW-7Ab*
Qian102032-8	27.98	39.92	38.75	*TaTGW-7Ab*
Suiningtuotuomai	25.71	31.39	34.36	*TaTGW-7Ab*
Waitoubai	24.06	27.20	26.95	*TaTGW-7Ab*
Wangshuibai	36.92	37.20	37.76	*TaTGW-7Ab*
Wankenmai081	37.04	38.09	41.05	*TaTGW-7Ab*
Xin19	35.56	39.16	41.74	*TaTGW-7Ab*
Xinmai0208	35.94	43.15	45.11	*TaTGW-7Ab*
Xinmai18	38.36	32.29	40.82	*TaTGW-7Ab*
Yifeng703	40.28	39.29	42.27	*TaTGW-7Ab*
Yu09113	38.76	40.12	43.27	*TaTGW-7Ab*
Yuguo	26.95	28.38	30.97	*TaTGW-7Ab*
Zhengmai366	33.55	35.67	39.51	*TaTGW-7Ab*
Zhengmai3666	39.05	37.67	40.52	*TaTGW-7Ab*
Zhongmai1139	52.09	47.31	52.39	*TaTGW-7Ab*
Zitongnvermai	22.03	28.40	28.58	*TaTGW-7Ab*

Table S3 Grain traits and allelic variations of 257 Chinese wheat mini-core collections (Pop 3)

Accession	2015			*TaTGW-7A* allele
	TGW(g)	Grain length(mm)	Grain width(mm)	
AC Phil	32.80	6.33	2.96	*TaTGW-7Aa*
Amazon	44.93	6.91	3.26	*TaTGW-7Aa*
Aodesa3	14.10	6.38	2.81	*TaTGW-7Aa*
Aongda183	33.48	6.14	3.21	*TaTGW-7Aa*
Atlas 66	34.84	7.20	3.16	*TaTGW-7Aa*
Baibiansui	26.87	5.88	2.94	*TaTGW-7Aa*
Baidongmai	30.67	6.87	2.75	*TaTGW-7Aa*
Baimangmai	22.76	5.52	2.77	*TaTGW-7Aa*
Bainong3217	41.17	6.75	3.48	*TaTGW-7Aa*
Baipu	34.12	6.17	3.09	*TaTGW-7Aa*
Baiqitou	38.06	7.28	3.23	*TaTGW-7Aa*
Baiyoumai	26.34	6.36	2.83	*TaTGW-7Aa*
Beijing8	40.00	6.35	3.37	*TaTGW-7Aa*
Biantouguangkeke	24.85	5.97	3.08	*TaTGW-7Aa*
Bihongsui	13.86	6.15	2.72	*TaTGW-7Aa*
Bima1	35.86	6.06	3.33	*TaTGW-7Aa*
Bimai26	41.11	7.35	3.38	*TaTGW-7Aa*
Bolero	28.42	8.68	3.88	*TaTGW-7Aa*
Changzhi6406	45.37	7.09	3.53	*TaTGW-7Aa*
Chengduguangtou	35.54	6.46	3.17	*TaTGW-7Aa*
Chixiaomai	39.72	6.93	3.40	*TaTGW-7Aa*
Chunmai	29.93	6.39	3.03	*TaTGW-7Aa*
Congyanghongmai1	30.23	6.42	3.24	*TaTGW-7Aa*
Dabaimai	36.34	6.32	3.15	*TaTGW-7Aa*
Dabaipi	23.11	6.07	2.57	*TaTGW-7Aa*
Dabaitou	27.28	5.80	3.21	*TaTGW-7Aa*
Dahongpi	45.77	7.66	3.17	*TaTGW-7Aa*
Dakoumai	25.47	5.74	2.81	*TaTGW-7Aa*
Daqingmang	18.47	5.85	2.45	*TaTGW-7Aa*
Dingxi24	30.54	7.14	3.23	*TaTGW-7Aa*
Dingxingzhai	33.28	6.01	2.84	*TaTGW-7Aa*
Dixiuzao	46.20	6.67	3.32	*TaTGW-7Aa*
Dongfanghong3	30.39	6.45	3.09	*TaTGW-7Aa*
Dongnong101	23.22	5.64	2.97	*TaTGW-7Aa*
Dunhuachunmai	18.50	5.96	2.38	*TaTGW-7Aa*
Early Premium	36.50	6.59	3.08	*TaTGW-7Aa*
Efu	32.51	6.55	3.24	*TaTGW-7Aa*
Efu	37.43	6.36	3.15	*TaTGW-7Aa*
Emai6	37.11	6.46	3.31	*TaTGW-7Aa*

ENESCO	42.81	5.91	2.86	*TaTGW-7Aa*
Enmai4	44.87	6.81	3.50	*TaTGW-7Aa*
Fengkang2	42.24	6.49	3.39	*TaTGW-7Aa*
Fengmai11	37.59	6.74	3.09	*TaTGW-7Aa*
Galaohan	22.56	6.86	2.77	*TaTGW-7Aa*
Gaojiasuo	30.20	6.06	3.11	*TaTGW-7Aa*
GB1(Jing97-995-3)	41.03	7.40	3.21	*TaTGW-7Aa*
GB4	46.93	6.68	3.60	*TaTGW-7Aa*
GB6	40.64	6.56	3.30	*TaTGW-7Aa*
GB8	42.20	6.34	3.40	*TaTGW-7Aa*
GB9	42.77	6.96	3.37	*TaTGW-7Aa*
Gejiaxiang	22.31	5.52	2.47	*TaTGW-7Aa*
Guangtou	22.11	5.60	2.41	*TaTGW-7Aa*
Guinong10	40.15	6.74	3.33	*TaTGW-7Aa*
Heshangmai	27.33	6.05	2.89	*TaTGW-7Aa*
Hongchunmai	29.25	5.95	2.49	*TaTGW-7Aa*
Hongdongmai	34.00	6.91	2.93	*TaTGW-7Aa*
Hongjinbaoyin	33.70	6.49	3.20	*TaTGW-7Aa*
Honglidangnianlao	30.42	5.87	3.02	*TaTGW-7Aa*
Hongmangmai	39.29	6.48	3.51	*TaTGW-7Aa*
Hongmangzi	28.89	6.86	3.42	*TaTGW-7Aa*
Hongpixiaomai	18.41	5.76	2.35	*TaTGW-7Aa*
Hongtuzi	30.36	6.63	3.06	*TaTGW-7Aa*
Huadong6	34.35	5.85	3.20	*TaTGW-7Aa*
Huining10	32.16	6.46	2.93	*TaTGW-7Aa*
Huoliaomai	20.76	5.88	2.72	*TaTGW-7Aa*
Jiangdongmen	28.94	6.07	3.03	*TaTGW-7Aa*
Jiangdongmen	32.48	6.05	3.03	*TaTGW-7Aa*
Jichun1016	37.11	6.62	3.14	*TaTGW-7Aa*
Jinan17	34.72	6.98	3.28	*TaTGW-7Aa*
Jinan2	44.03	6.63	3.46	*TaTGW-7Aa*
Jingxizao	27.24	7.47	3.45	*TaTGW-7Aa*
Jinhuangmai	28.43	6.55	2.82	*TaTGW-7Aa*
Jinmai2148	44.37	6.83	3.35	*TaTGW-7Aa*
Jinmai4	25.38	5.60	2.98	*TaTGW-7Aa*
Kangdingxiaomai	25.58	6.50	3.08	*TaTGW-7Aa*
Kangxiu10	35.10	6.26	3.30	*TaTGW-7Aa*
Kefeng3	19.20	6.11	3.14	*TaTGW-7Aa*
Kelao4	36.14	6.82	3.34	*TaTGW-7Aa*
Keshi1	16.22	5.53	2.45	*TaTGW-7Aa*
Keshibaipi	30.36	6.31	2.70	*TaTGW-7Aa*
Laizhou953	33.10	6.84	3.35	*TaTGW-7Aa*
Laizhou953	43.19	6.00	3.14	*TaTGW-7Aa*
Lankao86(79)1-2-7	64.26	7.31	3.47	*TaTGW-7Aa*

Laomai	36.24	6.26	3.06	*TaTGW-7Aa*
Laoqimai	32.01	6.05	2.92	*TaTGW-7Aa*
Liuyuehuang	25.90	7.09	3.15	*TaTGW-7Aa*
Lovrin 10	38.38	6.64	3.32	*TaTGW-7Aa*
Lumai1	39.06	6.67	3.21	*TaTGW-7Aa*
Lumai19	50.90	7.01	3.38	*TaTGW-7Aa*
Lvhan328	50.26	7.36	3.66	*TaTGW-7Aa*
Mangxiaomai	18.10	6.23	2.64	*TaTGW-7Aa*
Meiqianwu	28.82	6.05	2.89	*TaTGW-7Aa*
Motuoxiaomai	27.68	6.34	2.93	*TaTGW-7Aa*
Nanda2419	38.49	6.43	2.82	*TaTGW-7Aa*
Neimai11	36.64	7.15	3.38	*TaTGW-7Aa*
Neixiang5	45.55	7.12	3.52	*TaTGW-7Aa*
Ningxia4	34.34	6.37	3.41	*TaTGW-7Aa*
Nongda139	33.96	6.90	2.93	*TaTGW-7Aa*
Nongda311	30.81	6.44	2.99	*TaTGW-7Aa*
Nonglin10	37.65	6.52	3.20	*TaTGW-7Aa*
Nuomai	32.06	6.50	3.20	*TaTGW-7Aa*
Ousang	41.42	7.01	3.21	*TaTGW-7Aa*
paozimai	38.81	6.71	3.31	*TaTGW-7Aa*
Pindong34	64.42	7.87	3.42	*TaTGW-7Aa*
Pindong904047-9	63.84	8.53	3.70	*TaTGW-7Aa*
Pingyuan50	32.18	5.87	3.20	*TaTGW-7Aa*
Qianjiaomai	34.77	6.48	2.90	*TaTGW-7Aa*
Rikeze8	39.43	6.14	3.19	*TaTGW-7Aa*
SAGITTRIO	36.48	6.49	2.99	*TaTGW-7Aa*
SANGIACOMO	39.36	6.41	3.02	*TaTGW-7Aa*
Shanglinxiaomai	22.11	5.79	2.68	*TaTGW-7Aa*
Shangluo81(2)4-6-6	52.45	8.27	3.77	*TaTGW-7Aa*
Shanmai	30.17	6.59	2.87	*TaTGW-7Aa*
Shanmai	30.49	7.05	2.72	*TaTGW-7Aa*
Shannong7859	42.60	7.16	3.36	*TaTGW-7Aa*
Shanzao8675	47.44	6.17	3.26	*TaTGW-7Aa*
Shengen	30.07	5.94	2.98	*TaTGW-7Aa*
Shijiazhuang407	34.48	5.54	2.89	*TaTGW-7Aa*
Shijiazhuang54	37.69	6.35	3.33	*TaTGW-7Aa*
Shite14	25.83	5.65	2.82	*TaTGW-7Aa*
Shuwan8	35.15	6.89	3.25	*TaTGW-7Aa*
St 2422/464	42.02	7.29	3.25	*TaTGW-7Aa*
Sumai3	39.06	6.39	3.28	*TaTGW-7Aa*
Sumai3	38.07	6.32	3.19	*TaTGW-7Aa*
SW605	43.76	5.88	2.72	*TaTGW-7Aa*
Taishan1	41.18	6.68	3.32	*TaTGW-7Aa*
Taizhong23	34.78	6.57	3.15	*TaTGW-7Aa*

Tanori	40.25	6.95	3.07	*TaTGW-7Aa*
Triumph	29.40	6.27	2.79	*TaTGW-7Aa*
Tuokexun1	23.77	6.21	2.60	*TaTGW-7Aa*
VAIOLET	33.93	6.09	3.35	*TaTGW-7Aa*
Wenmai6	44.19	6.48	3.33	*TaTGW-7Aa*
Wujiangzhuo	19.10	6.61	2.67	*TaTGW-7Aa*
Wumangchunmai	12.15	6.73	2.75	*TaTGW-7Aa*
Wumangchunmai	24.51	5.68	2.97	*TaTGW-7Aa*
wuyuanmai	29.72	6.26	3.09	*TaTGW-7Aa*
Xiannong39	45.01	7.18	3.32	*TaTGW-7Aa*
Xiaohongpi	27.66	6.07	2.72	*TaTGW-7Aa*
Xiaomaibai	25.08	6.26	2.76	*TaTGW-7Aa*
Xiaoyan6	37.19	6.67	3.12	*TaTGW-7Aa*
Xindong2	33.39	6.09	3.22	*TaTGW-7Aa*
Xingyi4	47.75	7.64	3.37	*TaTGW-7Aa*
Xinkexing9	21.55	5.80	2.46	*TaTGW-7Aa*
Xinshuguang1	42.44	6.76	3.19	*TaTGW-7Aa*
Xinshuguang6	31.67	6.72	3.35	*TaTGW-7Aa*
Yanan11	21.43	7.37	2.77	*TaTGW-7Aa*
Yangmai	16.39	6.29	2.72	*TaTGW-7Aa*
Yangmai	21.47	5.70	2.66	*TaTGW-7Aa*
Yangmai158	42.37	6.85	3.36	*TaTGW-7Aa*
Yannong15	30.88	5.22	3.22	*TaTGW-7Aa*
Yanzhan1	43.25	7.05	3.59	*TaTGW-7Aa*
Yimai2	43.01	6.64	3.31	*TaTGW-7Aa*
youbao	42.45	6.91	3.63	*TaTGW-7Aa*
Youzimai	28.15	5.41	2.74	*TaTGW-7Aa*
Yuandong822	45.70	6.72	3.47	*TaTGW-7Aa*
Yunmai34	42.73	6.98	3.38	*TaTGW-7Aa*
Yuqiumai	32.48	5.96	3.02	*TaTGW-7Aa*
Zangdong4	33.22	6.66	3.02	*TaTGW-7Aa*
Zaosui30	37.58	6.06	3.45	*TaTGW-7Aa*
Zaoxiaomai	32.82	5.92	3.20	*TaTGW-7Aa*
Zhenghua0840-3	36.91	6.78	3.48	*TaTGW-7Aa*
Zhengzhou6	39.13	6.92	3.49	*TaTGW-7Aa*
Zhengzhou741	26.86	6.69	2.87	*TaTGW-7Aa*
Zhongyou9507	49.57	7.29	3.38	*TaTGW-7Aa*
Zhugoumai	24.88	6.48	3.09	*TaTGW-7Aa*
Zijiehong	30.58	6.62	3.10	*TaTGW-7Aa*
Zipi	25.02	6.21	2.66	*TaTGW-7Aa*
Aifeng3	33.27	6.13	3.15	*TaTGW-7Ab*
Am3	38.85	7.29	3.08	*TaTGW-7Ab*
Am9	24.95	5.63	2.54	*TaTGW-7Ab*
Anhui3	32.97	6.04	3.24	*TaTGW-7Ab*

Baihuamai	22.77	5.44	2.65	*TaTGW-7Ab*
Baihuomai	21.45	5.07	2.71	*TaTGW-7Ab*
Baimaizi	26.41	5.61	2.88	*TaTGW-7Ab*
Baimangmai	21.33	6.02	2.64	*TaTGW-7Ab*
Baimangmai	15.30	5.60	2.70	*TaTGW-7Ab*
Baimazha	18.93	5.79	3.15	*TaTGW-7Ab*
Baiqimai	28.73	6.02	2.83	*TaTGW-7Ab*
Baiqimmai	23.04	5.87	2.76	*TaTGW-7Ab*
Baitiaoyu	22.58	5.72	2.88	*TaTGW-7Ab*
Baituzimai	17.24	5.21	2.42	*TaTGW-7Ab*
Banjiemang	25.76	5.64	2.63	*TaTGW-7Ab*
Bianbachunmai-6	29.74	6.17	2.87	*TaTGW-7Ab*
Bima4	38.18	6.25	3.33	*TaTGW-7Ab*
Changmangshibiantou	26.42	5.72	2.80	*TaTGW-7Ab*
Cheqianzi	34.02	6.73	3.06	*TaTGW-7Ab*
Chushanbao	30.20	5.48	2.87	*TaTGW-7Ab*
CS	30.48	6.33	3.14	*TaTGW-7Ab*
Dabaimai	25.94	5.89	2.57	*TaTGW-7Ab*
Daimanghongmai	24.25	6.27	2.78	*TaTGW-7Ab*
Dalibanmang	29.72	5.74	2.92	*TaTGW-7Ab*
Dayuhua	23.62	5.49	2.86	*TaTGW-7Ab*
Fan6	36.10	5.97	3.04	*TaTGW-7Ab*
Fan6	37.76	6.06	3.14	*TaTGW-7Ab*
Fengchan3	36.54	6.35	3.31	*TaTGW-7Ab*
Fumai	28.78	5.80	2.76	*TaTGW-7Ab*
Fuzhuang30	27.60	5.70	3.01	*TaTGW-7Ab*
Gaoyuan506	27.10	6.61	3.25	*TaTGW-7Ab*
GB10	49.57	7.05	3.61	*TaTGW-7Ab*
GB2	42.70	6.48	3.42	*TaTGW-7Ab*
GB3	43.49	6.58	3.61	*TaTGW-7Ab*
GB5	41.32	6.75	3.53	*TaTGW-7Ab*
GB7	33.41	6.20	3.05	*TaTGW-7Ab*
Hanzhongbai	23.91	5.83	2.86	*TaTGW-7Ab*
Hhike	32.43	6.22	3.10	*TaTGW-7Ab*
Honggoudou	31.41	5.16	3.13	*TaTGW-7Ab*
Honghuamai	31.77	6.56	3.35	*TaTGW-7Ab*
Honghuazao	21.84	5.75	2.84	*TaTGW-7Ab*
Hongjinmai	21.54	5.47	2.68	*TaTGW-7Ab*
Hongjuanmang	29.17	5.57	2.75	*TaTGW-7Ab*
Honglaomai	19.20	5.65	2.37	*TaTGW-7Ab*
Hongmai	27.52	6.01	2.96	*TaTGW-7Ab*
Hongpidongmai	18.55	6.13	2.49	*TaTGW-7Ab*
Hongqiangcheng	25.28	5.64	2.88	*TaTGW-7Ab*
Hongxumai	22.62	6.17	2.70	*TaTGW-7Ab*

Huangguaxian	25.78	5.97	2.70	*TaTGW-7Ab*
Huangshuibai	31.37	5.71	3.04	*TaTGW-7Ab*
Huoliyan	29.67	6.20	2.75	*TaTGW-7Ab*
Huomai	17.04	5.27	2.35	*TaTGW-7Ab*
Jiahongmai	22.52	5.64	2.72	*TaTGW-7Ab*
Jiangmai	31.18	5.86	2.93	*TaTGW-7Ab*
Jianmai	14.04	5.79	2.48	*TaTGW-7Ab*
Jingyang60	23.97	5.26	2.80	*TaTGW-7Ab*
Jinmai3	41.45	6.47	3.28	*TaTGW-7Ab*
Lanhuamai	24.99	5.25	2.82	*TaTGW-7Ab*
Laogudian	24.49	5.16	2.57	*TaTGW-7Ab*
Laolaixia	28.02	6.19	2.83	*TaTGW-7Ab*
Laotutou	17.40	6.24	2.64	*TaTGW-7Ab*
Lengtiaohongmai	18.19	5.95	2.55	*TaTGW-7Ab*
Lianglaiyoubaipixiaomai	22.94	5.75	2.76	*TaTGW-7Ab*
Liuzhutou	30.39	6.24	3.12	*TaTGW-7Ab*
Liying5	46.14	6.81	3.55	*TaTGW-7Ab*
Mahuaban	16.77	5.28	2.22	*TaTGW-7Ab*
Mazhamai	26.56	5.37	2.78	*TaTGW-7Ab*
Mingxian169	26.20	6.61	2.92	*TaTGW-7Ab*
Niuzhijia	24.95	6.61	2.55	*TaTGW-7Ab*
Qiangchangmai	23.49	5.43	2.82	*TaTGW-7Ab*
Sanyuehuang	23.57	5.46	2.70	*TaTGW-7Ab*
Sanyuehuang	24.72	5.84	2.77	*TaTGW-7Ab*
Shanxibaimai	16.38	5.48	2.61	*TaTGW-7Ab*
Shuilizhan	28.77	6.08	3.00	*TaTGW-7Ab*
Shuiyuan86	24.36	6.23	2.68	*TaTGW-7Ab*
Songxinmai	35.20	6.09	3.16	*TaTGW-7Ab*
Tongjiabeixiaomai	28.73	6.07	2.96	*TaTGW-7Ab*
Tumangmai	23.90	5.61	3.04	*TaTGW-7Ab*
Wangshuibai	32.55	6.74	3.01	*TaTGW-7Ab*
Xianmai	26.88	5.92	2.90	*TaTGW-7Ab*
Xiaofoshou	20.58	5.02	2.60	*TaTGW-7Ab*
Xiaokouhong	29.08	6.18	2.94	*TaTGW-7Ab*
Xiaosanyuehuang	24.84	5.90	2.81	*TaTGW-7Ab*
Xishanbiansui	24.37	5.60	3.03	*TaTGW-7Ab*
Youmangbaifu	21.01	6.05	2.58	*TaTGW-7Ab*
Youmangsaogudan	21.26	5.60	2.82	*TaTGW-7Ab*
Zhemai1	27.32	6.35	3.10	*TaTGW-7Ab*
Zhongguochun	28.83	6.05	3.10	*TaTGW-7Ab*
Zhuoludongmai	28.46	6.10	2.74	*TaTGW-7Ab*
Zhushimai	27.22	6.08	2.74	*TaTGW-7Ab*

Table S4 Allelic variation and geographic distribution for 501 accessions (Pop 4)

Accession	*TaTGW-7A* allele	Agroecological region	Accession	*TaTGW-7A* allele	Agroecological region
03-885	*TaTGW-7Aa*	I	Yan2415	*TaTGW-7Aa*	II
03G7	*TaTGW-7Aa*	I	Yan2801	*TaTGW-7Aa*	II
99G80	*TaTGW-7Aa*	I	Yan99-5	*TaTGW-7Aa*	II
AR2	*TaTGW-7Aa*	I	Yanfu188	*TaTGW-7Aa*	II
Baimanghong	*TaTGW-7Aa*	I	Yannong19	*TaTGW-7Aa*	II
Baofeng10-82	*TaTGW-7Aa*	I	Yannong24	*TaTGW-7Aa*	II
Baomai3	*TaTGW-7Aa*	I	Yannong24	*TaTGW-7Aa*	II
Baomai8	*TaTGW-7Aa*	I	Yanzhan4110	*TaTGW-7Aa*	II
CA0178	*TaTGW-7Aa*	I	Youbao	*TaTGW-7Aa*	II
CA9532	*TaTGW-7Aa*	I	Youzimai	*TaTGW-7Aa*	II
CA9641	*TaTGW-7Aa*	I	Yumai13	*TaTGW-7Aa*	II
Cang97-051	*TaTGW-7Aa*	I	Yumai18	*TaTGW-7Aa*	II
Cangmai119	*TaTGW-7Aa*	I	Yumai2	*TaTGW-7Aa*	II
Dongfanghong3	*TaTGW-7Aa*	I	Yumai2	*TaTGW-7Aa*	II
Gaocheng8901	*TaTGW-7Aa*	I	Yumai25	*TaTGW-7Aa*	II
Gaomai119	*TaTGW-7Aa*	I	Yumai34	*TaTGW-7Aa*	II
Gaoyou9409	*TaTGW-7Aa*	I	Yumai35	*TaTGW-7Aa*	II
Gaoyou9415	*TaTGW-7Aa*	I	Yumai47	*TaTGW-7Aa*	II
Gaoyou9618	*TaTGW-7Aa*	I	Yumai49	*TaTGW-7Aa*	II
Guan35	*TaTGW-7Aa*	I	Yumai54	*TaTGW-7Aa*	II
Han4564	*TaTGW-7Aa*	I	Yumai62	*TaTGW-7Aa*	II
Han5030	*TaTGW-7Aa*	I	Yun97169	*TaTGW-7Aa*	II
Han9565	*TaTGW-7Aa*	I	Yunong69	*TaTGW-7Aa*	II
Heguan35	*TaTGW-7Aa*	I	Zheng9023	*TaTGW-7Aa*	II
Heng4338	*TaTGW-7Aa*	I	Zhengmai9023	*TaTGW-7Aa*	II
Heng6149	*TaTGW-7Aa*	I	Zhengmai9405	*TaTGW-7Aa*	II
Heng7228	*TaTGW-7Aa*	I	Zhengmai98	*TaTGW-7Aa*	II
Hengyou18	*TaTGW-7Aa*	I	Zhengnong19	*TaTGW-7Aa*	II
Henong326	*TaTGW-7Aa*	I	Zhengyin1	*TaTGW-7Aa*	II
Henong638	*TaTGW-7Aa*	I	Zhengyumai518	*TaTGW-7Aa*	II
Henong825	*TaTGW-7Aa*	I	Zhengzhou974	*TaTGW-7Aa*	II
Henong972	*TaTGW-7Aa*	I	Zhengzhou992	*TaTGW-7Aa*	II
Henongkangbai4	*TaTGW-7Aa*	I	Zhongyou989	*TaTGW-7Aa*	II
Ji95-6023	*TaTGW-7Aa*	I	Zhongyu1095	*TaTGW-7Aa*	II
Jimai33	*TaTGW-7Aa*	I	Zhongyu5	*TaTGW-7Aa*	II
Jimai50	*TaTGW-7Aa*	I	Zhou92031	*TaTGW-7Aa*	II
Jimai67	*TaTGW-7Aa*	I	Zhou98165	*TaTGW-7Aa*	II
Jingdong8	*TaTGW-7Aa*	I	Zhoumai11	*TaTGW-7Aa*	II
Jinghe951	*TaTGW-7Aa*	I	Zhoumai16	*TaTGW-7Aa*	II
Jingnong98-100	*TaTGW-7Aa*	I	Zhoumai17	*TaTGW-7Aa*	II

Jinmai31	*TaTGW-7Aa*	I	Zhoumai18	*TaTGW-7Aa*	II
Jinmai73	*TaTGW-7Aa*	I	Zhoumai18	*TaTGW-7Aa*	II
Linfen127	*TaTGW-7Aa*	I	Zhoumai19	*TaTGW-7Aa*	II
Linfen137	*TaTGW-7Aa*	I	Zhoumai20	*TaTGW-7Aa*	II
Linfen139	*TaTGW-7Aa*	I	Zhoumai23	*TaTGW-7Aa*	II
Linken2	*TaTGW-7Aa*	I	Zhoumai25	*TaTGW-7Aa*	II
Luofulin10	*TaTGW-7Aa*	I	Zhoumai31	*TaTGW-7Aa*	II
Luofulin13	*TaTGW-7Aa*	I	Zi0706	*TaTGW-7Aa*	II
Nongda116	*TaTGW-7Aa*	I	Annong1116	*TaTGW-7Ab*	II
Nongda123	*TaTGW-7Aa*	I	Fengdecunmai5	*TaTGW-7Ab*	II
Nongda139	*TaTGW-7Aa*	I	Huaimai0360	*TaTGW-7Ab*	II
Nongda3214	*TaTGW-7Aa*	I	Huangguaxian SP11	*TaTGW-7Ab*	II
Nongda3291	*TaTGW-7Aa*	I	Huapei8	*TaTGW-7Ab*	II
Nongda3395	*TaTGW-7Aa*	I	Luo2267	*TaTGW-7Ab*	II
R146	*TaTGW-7Aa*	I	Shan253	*TaTGW-7Ab*	II
Shanqianmai2	*TaTGW-7Aa*	I	Shan512	*TaTGW-7Ab*	II
Shiami15	*TaTGW-7Aa*	I	Shannong981	*TaTGW-7Ab*	II
Shihao02-1	*TaTGW-7Aa*	I	Shanyou225	*TaTGW-7Ab*	II
Shijiazhuang8	*TaTGW-7Aa*	I	WaitoubaiSP17	*TaTGW-7Ab*	II
Shimai12	*TaTGW-7Aa*	I	Wannongken081	*TaTGW-7Ab*	II
Shimai14	*TaTGW-7Aa*	I	Xin19	*TaTGW-7Ab*	II
Shimai16	*TaTGW-7Aa*	I	Xinmai0208	*TaTGW-7Ab*	II
Shixin618	*TaTGW-7Aa*	I	Xinmai18	*TaTGW-7Ab*	II
Shixin703	*TaTGW-7Aa*	I	Xinong12208019	*TaTGW-7Ab*	II
Shixin733	*TaTGW-7Aa*	I	Yan475	*TaTGW-7Ab*	II
Shiyou17	*TaTGW-7Aa*	I	Yimai26	*TaTGW-7Ab*	II
Tai10604	*TaTGW-7Aa*	I	Yumai50	*TaTGW-7Ab*	II
X9610	*TaTGW-7Aa*	I	Yumai7	*TaTGW-7Ab*	II
Xiaoyan6	*TaTGW-7Aa*	I	Zhengmai366	*TaTGW-7Ab*	II
Xing05-4241	*TaTGW-7Aa*	I	Zhengmai3666	*TaTGW-7Ab*	II
Xingmai13	*TaTGW-7Aa*	I	Zhoumai13	*TaTGW-7Ab*	II
Xingmai6	*TaTGW-7Aa*	I	02P67	*TaTGW-7Aa*	III
Xinong622	*TaTGW-7Aa*	I	02Y151	*TaTGW-7Aa*	III
Xinong889	*TaTGW-7Aa*	I	An96-8	*TaTGW-7Aa*	III
Xinong979	*TaTGW-7Aa*	I	Annong0942	*TaTGW-7Aa*	III
Yi5265	*TaTGW-7Aa*	I	Annong91168	*TaTGW-7Aa*	III
Yi5385	*TaTGW-7Aa*	I	Annong94022	*TaTGW-7Aa*	III
Yi95-5219	*TaTGW-7Aa*	I	Annong98005	*TaTGW-7Aa*	III
Yimai34	*TaTGW-7Aa*	I	CP02-63-13-1	*TaTGW-7Aa*	III
Yishi02-1	*TaTGW-7Aa*	I	CP02-8-5-6-1	*TaTGW-7Aa*	III
Zhongmai1187	*TaTGW-7Aa*	I	CP02-9-3-1-1-1	*TaTGW-7Aa*	III
Zhongmai155	*TaTGW-7Aa*	I	CP20-39-11-1	*TaTGW-7Aa*	III
Zhongmai895	*TaTGW-7Aa*	I	E86642	*TaTGW-7Aa*	III

Zhongyou9507	*TaTGW-7Aa*	I	E91727	*TaTGW-7Aa*	III
Chadianhongmai	*TaTGW-7Ab*	I	E’en1	*TaTGW-7Aa*	III
Gaoyou1817	*TaTGW-7Ab*	I	Emai6	*TaTGW-7Aa*	III
Heng87-6476	*TaTGW-7Ab*	I	Guandong107	*TaTGW-7Aa*	III
Heng97-4119	*TaTGW-7Ab*	I	Hongnong1	*TaTGW-7Aa*	III
Jingdong10	*TaTGW-7Ab*	I	M010	*TaTGW-7Aa*	III
Jinhe0459	*TaTGW-7Ab*	I	M013	*TaTGW-7Aa*	III
Jinmai60	*TaTGW-7Ab*	I	M015	*TaTGW-7Aa*	III
Jinnong215	*TaTGW-7Ab*	I	M019	*TaTGW-7Aa*	III
Yifeng703	*TaTGW-7Ab*	I	M040	*TaTGW-7Aa*	III
Zhongmai1139	*TaTGW-7Ab*	I	M046	*TaTGW-7Aa*	III
984121	*TaTGW-7Aa*	II	M051	*TaTGW-7Aa*	III
Aifengzao8	*TaTGW-7Aa*	II	M094	*TaTGW-7Aa*	III
Aikang58	*TaTGW-7Aa*	II	M108	*TaTGW-7Aa*	III
Aizao64	*TaTGW-7Aa*	II	M126	*TaTGW-7Aa*	III
Annong0807	*TaTGW-7Aa*	II	Ning9548	*TaTGW-7Aa*	III
Annong0932	*TaTGW-7Aa*	II	Ning97-18	*TaTGW-7Aa*	III
Annong0942-13	*TaTGW-7Aa*	II	Ning97-41	*TaTGW-7Aa*	III
Annong1001	*TaTGW-7Aa*	II	Ning99415-8	*TaTGW-7Aa*	III
Annong1014	*TaTGW-7Aa*	II	Ningmai9	*TaTGW-7Aa*	III
Annong1039	*TaTGW-7Aa*	II	Shen32109	*TaTGW-7Aa*	III
Annong1106	*TaTGW-7Aa*	II	Sumai3	*TaTGW-7Aa*	III
Annong1107	*TaTGW-7Aa*	II	Sumai3	*TaTGW-7Aa*	III
Annong1108	*TaTGW-7Aa*	II	Sumai6	*TaTGW-7Aa*	III
Annong1110	*TaTGW-7Aa*	II	Wangshuibai	*TaTGW-7Aa*	III
Annong1114	*TaTGW-7Aa*	II	Wanmai33	*TaTGW-7Aa*	III
Annong8455	*TaTGW-7Aa*	II	Wen2540	*TaTGW-7Aa*	III
Annong92484W	*TaTGW-7Aa*	II	Wumai1	*TaTGW-7Aa*	III
Annong9267	*TaTGW-7Aa*	II	Y14	*TaTGW-7Aa*	III
Bainong207	*TaTGW-7Aa*	II	Y18	*TaTGW-7Aa*	III
Bainong3271(pm 13)	*TaTGW-7Aa*	II	Yang97-65	*TaTGW-7Aa*	III
Bainong64	*TaTGW-7Aa*	II	Yangmai10	*TaTGW-7Aa*	III
Bainongaikang58	*TaTGW-7Aa*	II	Yangmai13	*TaTGW-7Aa*	III
Baipi224 (SP19)	*TaTGW-7Aa*	II	Yangmai158	*TaTGW-7Aa*	III
Baitutou(SP15)	*TaTGW-7Aa*	II	Yangmai16	*TaTGW-7Aa*	III
Baiyuhua(SP16)	*TaTGW-7Aa*	II	Yangmai19	*TaTGW-7Aa*	III
Bolunxuan182(LC-33)	*TaTGW-7Aa*	II	Yangmai20	*TaTGW-7Aa*	III
Dangmai2	*TaTGW-7Aa*	II	Yangmai3	*TaTGW-7Aa*	III
Danshi802	*TaTGW-7Aa*	II	Yangmai4	*TaTGW-7Aa*	III
Dinghong208	*TaTGW-7Aa*	II	Yangmai9	*TaTGW-7Aa*	III
Fanmai5	*TaTGW-7Aa*	II	Yangnuomai1	*TaTGW-7Aa*	III
Fanmai8	*TaTGW-7Aa*	II	Baihuomai SP10	*TaTGW-7Ab*	III

Fu0382(LB-5)	*TaTGW-7Aa*	II	E158	*TaTGW-7Ab*	III
Guomai0608	*TaTGW-7Aa*	II	E66378	*TaTGW-7Ab*	III
Guomai10	*TaTGW-7Aa*	II	E81027	*TaTGW-7Ab*	III
Guomai8	*TaTGW-7Aa*	II	M104	*TaTGW-7Ab*	III
Guoshengmai1(LA-30)	*TaTGW-7Aa*	II	Wangshuibai(SP9)	*TaTGW-7Ab*	III
Hongwan3	*TaTGW-7Aa*	II	Yangmai12	*TaTGW-7Ab*	III
Huaimai0320	*TaTGW-7Aa*	II	Ailiduo	*TaTGW-7Aa*	IV
Huaimai0882	*TaTGW-7Aa*	II	Chuan96003	*TaTGW-7Aa*	IV
Huaishi0806(LC-15)	*TaTGW-7Aa*	II	Chuanmai107	*TaTGW-7Aa*	IV
Huapei0616H-119	*TaTGW-7Aa*	II	Chuanmai107	*TaTGW-7Aa*	IV
Huarui0712	*TaTGW-7Aa*	II	Chuanmai20	*TaTGW-7Aa*	IV
Hulutou(SP14)	*TaTGW-7Aa*	II	Chuanmai22	*TaTGW-7Aa*	IV
Ji5099	*TaTGW-7Aa*	II	Chuanmai28	*TaTGW-7Aa*	IV
Ji5219	*TaTGW-7Aa*	II	Chuanmai42	*TaTGW-7Aa*	IV
Ji95-6023	*TaTGW-7Aa*	II	Chuanyu12	*TaTGW-7Aa*	IV
Jimai035037	*TaTGW-7Aa*	II	Demai3	*TaTGW-7Aa*	IV
Jimai056487	*TaTGW-7Aa*	II	Ebo	*TaTGW-7Aa*	IV
Jimai19	*TaTGW-7Aa*	II	Fan6	*TaTGW-7Aa*	IV
Jimai19	*TaTGW-7Aa*	II	Fan7	*TaTGW-7Aa*	IV
Jimai20	*TaTGW-7Aa*	II	Fengmai24	*TaTGW-7Aa*	IV
Jimai20	*TaTGW-7Aa*	II	Guinong775	*TaTGW-7Aa*	IV
Jimai20	*TaTGW-7Aa*	II	Jing9308	*TaTGW-7Aa*	IV
Jimai24	*TaTGW-7Aa*	II	Jingmai11	*TaTGW-7Aa*	IV
Jimai36	*TaTGW-7Aa*	II	Kefeng14	*TaTGW-7Aa*	IV
Jimai38	*TaTGW-7Aa*	II	Mian2000-18	*TaTGW-7Aa*	IV
Jimai5319	*TaTGW-7Aa*	II	Mian2000-19	*TaTGW-7Aa*	IV
Jimai7251--1	*TaTGW-7Aa*	II	Mian2000-9	*TaTGW-7Aa*	IV
Jimai7251--2	*TaTGW-7Aa*	II	Miannong4	*TaTGW-7Aa*	IV
Jinan13	*TaTGW-7Aa*	II	Mianyang20	*TaTGW-7Aa*	IV
Jinan16	*TaTGW-7Aa*	II	Mianyang26	*TaTGW-7Aa*	IV
Jinan2	*TaTGW-7Aa*	II	Mianyang29	*TaTGW-7Aa*	IV
Jinfeng0459	*TaTGW-7Aa*	II	Mianyang960107	*TaTGW-7Aa*	IV
Jinfeng6164	*TaTGW-7Aa*	II	Mianyang98-17	*TaTGW-7Aa*	IV
Jining16	*TaTGW-7Aa*	II	Mianyang98-20	*TaTGW-7Aa*	IV
Jinli88	*TaTGW-7Aa*	II	N711	*TaTGW-7Aa*	IV
Junmai35	*TaTGW-7Aa*	II	Neimai10	*TaTGW-7Aa*	IV
Laizhou953	*TaTGW-7Aa*	II	Ourou	*TaTGW-7Aa*	IV
Lankao24	*TaTGW-7Aa*	II	Qian079984-14	*TaTGW-7Aa*	IV
Lankao298	*TaTGW-7Aa*	II	Qianmai18	*TaTGW-7Aa*	IV
Lankao906	*TaTGW-7Aa*	II	R111	*TaTGW-7Aa*	IV

Linhan619	*TaTGW-7Aa*	II	R122	*TaTGW-7Aa*	IV
Linmai2	*TaTGW-7Aa*	II	R131	*TaTGW-7Aa*	IV
Linyou1583	*TaTGW-7Aa*	II	R57	*TaTGW-7Aa*	IV
Longke0901	*TaTGW-7Aa*	II	S001	*TaTGW-7Aa*	IV
Lumai15	*TaTGW-7Aa*	II	Yaanzao	*TaTGW-7Aa*	IV
Lumai16	*TaTGW-7Aa*	II	Yu0926	*TaTGW-7Aa*	IV
Lumai21	*TaTGW-7Aa*	II	Yumai3	*TaTGW-7Aa*	IV
Lumai22	*TaTGW-7Aa*	II	Yumai7	*TaTGW-7Aa*	IV
Lumai23	*TaTGW-7Aa*	II	Chuanmai24	*TaTGW-7Ab*	IV
Lumai23	*TaTGW-7Aa*	II	Jingmai10	*TaTGW-7Ab*	IV
Lumai7	*TaTGW-7Aa*	II	Langzhongbaimaizi	*TaTGW-7Ab*	IV
Luo6099	*TaTGW-7Aa*	II	Mianmai37	*TaTGW-7Ab*	IV
Luo9920	*TaTGW-7Aa*	II	Mianyang940112	*TaTGW-7Ab*	IV
Luomai21--1	*TaTGW-7Aa*	II	Neimai11	*TaTGW-7Ab*	IV
Luomai21--2	*TaTGW-7Aa*	II	Neimai8	*TaTGW-7Ab*	IV
Luyuan502	*TaTGW-7Aa*	II	Peilingxuxumai	*TaTGW-7Ab*	IV
Mazhamai	*TaTGW-7Aa*	II	Qian102032-8	*TaTGW-7Ab*	IV
Mingtian0417	*TaTGW-7Aa*	II	Suiningtuotuommai	*TaTGW-7Ab*	IV
Neixiang188	*TaTGW-7Aa*	II	Yin11-12	*TaTGW-7Ab*	IV
Neixiang19	*TaTGW-7Aa*	II	Yu09113	*TaTGW-7Ab*	IV
Neixiang203	*TaTGW-7Aa*	II	Yunmai39	*TaTGW-7Ab*	IV
Neixiang36	*TaTGW-7Aa*	II	Yunmai42	*TaTGW-7Ab*	IV
Neixiang5	*TaTGW-7Aa*	II	Zitongnvermai	*TaTGW-7Ab*	IV
PH1521	*TaTGW-7Aa*	II	Heilongjiangzhongshi1	*TaTGW-7Aa*	VI
PH82-2-2	*TaTGW-7Aa*	II	Heilongjiangzhongshi11	*TaTGW-7Aa*	VI
PH85-1-1	*TaTGW-7Aa*	II	Heilongjiangzhongshi12	*TaTGW-7Aa*	VI
S038186	*TaTGW-7Aa*	II	Heilongjiangzhongshi13	*TaTGW-7Aa*	VI
Sanyuehuang	*TaTGW-7Aa*	II	Heilongjiangzhongshi15	*TaTGW-7Aa*	VI
Shan150	*TaTGW-7Aa*	II	Heilongjiangzhongshi2	*TaTGW-7Aa*	VI
Shan160	*TaTGW-7Aa*	II	Heilongjiangzhongshi3	*TaTGW-7Aa*	VI
Shan229	*TaTGW-7Aa*	II	Heilongjiangzhongshi4	*TaTGW-7Aa*	VI
Shan302518	*TaTGW-7Aa*	II	Heilongjiangzhongshi5	*TaTGW-7Aa*	VI

Shan354	*TaTGW-7Aa*	II	Heilongjiangzhongshi7	*TaTGW-7Aa*	VI
Shan715	*TaTGW-7Aa*	II	Heilongjiangzhongshi8	*TaTGW-7Aa*	VI
Shandongfu63	*TaTGW-7Aa*	II	Longfumai12	*TaTGW-7Aa*	VI
Shangqiuhuloutou	*TaTGW-7Aa*	II	Longfumai13	*TaTGW-7Aa*	VI
Shannong055843 (LB-27)	*TaTGW-7Aa*	II	Longfumai7	*TaTGW-7Aa*	VI
Shannong1355	*TaTGW-7Aa*	II	Longfumai8	*TaTGW-7Aa*	VI
Shannong15	*TaTGW-7Aa*	II	Longfumai9	*TaTGW-7Aa*	VI
Shannong413863	*TaTGW-7Aa*	II	Longmai12	*TaTGW-7Aa*	VI
Shannong617	*TaTGW-7Aa*	II	Longmai15	*TaTGW-7Aa*	VI
Shannong664	*TaTGW-7Aa*	II	Longmai19	*TaTGW-7Aa*	VI
Shannong7859	*TaTGW-7Aa*	II	Longmai26	*TaTGW-7Aa*	VI
Shi4185	*TaTGW-7Aa*	II	Heilongjiangzhongshi0	*TaTGW-7Ab*	VI
shi4185	*TaTGW-7Aa*	II	Longfumai14	*TaTGW-7Ab*	VI
Shijiazhuang54	*TaTGW-7Aa*	II	Longfumai2	*TaTGW-7Ab*	VI
Su553	*TaTGW-7Aa*	II	Longfumai3	*TaTGW-7Ab*	VI
Su853	*TaTGW-7Aa*	II	Longfumai4	*TaTGW-7Ab*	VI
Tai18	*TaTGW-7Aa*	II	Longfumai5	*TaTGW-7Ab*	VI
Taikong6	*TaTGW-7Aa*	II	Biyumai	*TaTGW-7Aa*	VIII
Taishan1	*TaTGW-7Aa*	II	Gan630	*TaTGW-7Aa*	VIII
Taishan23	*TaTGW-7Aa*	II	Ganmai8	*TaTGW-7Aa*	VIII
TuhulutouSP13	*TaTGW-7Aa*	II	Gelannne	*TaTGW-7Aa*	VIII
Wan7107	*TaTGW-7Aa*	II	Kefeng3	*TaTGW-7Aa*	VIII
Wanke06290	*TaTGW-7Aa*	II	Kefeng6	*TaTGW-7Aa*	VIII
Wanke08585	*TaTGW-7Aa*	II	Longchun20	*TaTGW-7Aa*	VIII
Wanke09636(LA-19)	*TaTGW-7Aa*	II	Longchun22	*TaTGW-7Aa*	VIII
Wanmai18	*TaTGW-7Aa*	II	Longchun8139	*TaTGW-7Aa*	VIII
Wanmai19	*TaTGW-7Aa*	II	Longchun9	*TaTGW-7Aa*	VIII
Wanmai38	*TaTGW-7Aa*	II	Ningchun16	*TaTGW-7Aa*	VIII
Wanmai38	*TaTGW-7Aa*	II	Ningchun18	*TaTGW-7Aa*	VIII
Wannong606	*TaTGW-7Aa*	II	Ningchun19	*TaTGW-7Aa*	VIII
Weimai18	*TaTGW-7Aa*	II	Ningchun30	*TaTGW-7Aa*	VIII
Wennong6	*TaTGW-7Aa*	II	Ningchun32	*TaTGW-7Aa*	VIII
Xian8	*TaTGW-7Aa*	II	Ningchun39	*TaTGW-7Aa*	VIII
Xiannong1	*TaTGW-7Aa*	II	Ningchun4	*TaTGW-7Aa*	VIII
Xiaoyan22	*TaTGW-7Aa*	II	Gaoyuan602	*TaTGW-7Ab*	VIII
Xiaoyan503	*TaTGW-7Aa*	II	Minqin732	*TaTGW-7Ab*	VIII
Xiaoyan54	*TaTGW-7Aa*	II	Ningchun37	*TaTGW-7Ab*	VIII
Xiaoyan921	*TaTGW-7Aa*	II	Qingchun533	*TaTGW-7Ab*	VIII

Xinmai0401	*TaTGW-7Aa*	II	Xinchun10	*TaTGW-7Aa*	X
Xinmai11	*TaTGW-7Aa*	II	Xinchun11	*TaTGW-7Aa*	X
Xinmai19023	*TaTGW-7Aa*	II	Xinchun13	*TaTGW-7Aa*	X
Xinmai23	*TaTGW-7Aa*	II	Xinchun6	*TaTGW-7Aa*	X
Xinmai9	*TaTGW-7Aa*	II	Xinchun7	*TaTGW-7Aa*	X
Xinong1163-20	*TaTGW-7Aa*	II	Xindong20	*TaTGW-7Aa*	X
Xinong1376	*TaTGW-7Aa*	II	Xindong24	*TaTGW-7Aa*	X
Xinong2611	*TaTGW-7Aa*	II	Xindong24	*TaTGW-7Aa*	X
Xinong291	*TaTGW-7Aa*	II	Xindong27	*TaTGW-7Aa*	X
Xinong6426	*TaTGW-7Aa*	II	Xindong28	*TaTGW-7Aa*	X
Xinyuanmai04130(LD-8)	*TaTGW-7Aa*	II	Xindong18	*TaTGW-7Ab*	X
Xu5034	*TaTGW-7Aa*	II	Xinmai12	*TaTGW-7Ab*	X
Xuke1	*TaTGW-7Aa*	II	Xinmai15	*TaTGW-7Ab*	X
Xumai25	*TaTGW-7Aa*	II	Xinmai16	*TaTGW-7Ab*	X
Xumai858	*TaTGW-7Aa*	II	Xinmai2	*TaTGW-7Ab*	X
Xumai9074	*TaTGW-7Aa*	II			

[a] I, Northern Winter Wheat Region; II, Yellow and Huai Valleys Winter Wheat Region; III, Middle and Lower; Yangtze River Valley Winter Wheat Region; IV, Southwestern Winter Wheat Region; VI, Northeastern Spring Wheat Region; VIII, Northwestern Spring Wheat Region; X, Xinjiang Winter-Spring Wheat Region

Table S6 Primers for quantitative RT-PCR, functional markers and amplification of the full-length gDNA and cDNA

Primer set	Primer sequence (5'-3')	Gene	Size of PCR fragments (bp)	Restriction enzyme
Q29	F: GCAAGCCCGTCAACATGTACTAC	*TaTGW-7A*	6667	
	R: AATTTCTGGACTTATGGGGAGCC			
Q35	F: TCTACCACGCAGCAAATCGCC	*TaTGW-7A*	6592	
	R: CTGGACTTATGGGGAGCCTCT			
Q37	F: GCCCGTCAACATGTACTACTC	*TaTGW-7A*	6617	
	R: GGTAGAGCTTCTCATAGCTGC			
29P	F: AAGGTTTGGGGAGTAAAGTTTT	*TaTGW-7A*	4394	
	R: AGTCAGAATTGTGCCTAAGTGC			
31P	F: GAGGGGTAGGATGAAGGAAGA	*TaTGW-7A*	4082	
	R: ATTTCTGGACTTATGGGGAGC			
SR	F: ACCCATCCCTCCATCCGTCG	*TaTGW-7A*	2336	
	R: ATCCGCCCACCTGGTAAAGC			
MQ	F: GCTACATACACGCACCAACCC	*TaTGW-7A*	250	*BsmAI*
	R: GACGAGAAGATAGGCGGACAG			
FQ	F: CAAGCTGGAGGAGCTGCTCTT	*TaTGW-7A*	156	
	R: TGGTACTGTTTTAGTTGTGGAGACATC			
Probe	FAM-CTCCGCCCCAAAGGTTAAAGTAAG-TAMARA			
Actin	F: AGCCATACTGTGCCAATC	*ACTIN*	134	
	R: GCAGTGGTGGTGAAGGAGTAA			
TG9	F: TGCATGTGGTATTGACGTAT	*TaTGW-7A*	204	
	R: GTTGTTCGGACAGAAAAGAG			
SLAF49035	F: TGGCTTCCACTGCTTGTGCG		390	*BstNI*
	R: CCCAACCGTTTCCCGTATCT			
SLAF28300	F: AGGACACCCCATACACACAC		220	*MluCI*
	R: CATTGCTCCACCTCGACTAC			
SLAF133263	F:CACCAAAACTGTCGTATCAT		425	*AluI*
	R:TGCTACTTTTCAAGGCTAGT			
SLAF6258	F:CTCAGCCTCCCTAACCAGAC		465	*AluI*
	R:CCCTTACACTAAAGGATCAAACAC			

Table S8 Significantly associated SLAF tags on genome of common wheat

Chromosome	SLAF number
1A	42
1B	50
1D	13
2A	118
2B	79
2D	12
3A	39
3B	32
3D	8
4A	45
4B	37
4D	13
5A	78
5B	75
5D	15
6A	55
6B	60
6D	18
7A	89
7B	50
7D	10
Total	938

Table S10 QTLs for TGW across five environments.

Chromosome	Flanking marker	LOD	R^2 (%)	Env.
1B	*Xgwm131-Xwmc419*	4.9-8.1	7.8-13.4	2008,2011,2012,2014,2015
3A1	*Xgwm162-Xcfa2134*	6.6-8.2	8.4-19.1	2008,2011,2012,2014,2015
3A2	*Xwmc112-Xgwm114*	4.5-6.9	6.5-12.7	2008,2011,2012,2014,2015
3D	*Xgwm114-Xcfd211*	4.1-6.5	5.7-10.1	2008,2011,2012,2014,2015
4B	*Xgwm192-2-Xgwm162*	4.9-9.7	7.2-19.8	2008,2011,2012,2014,2015
4D	*Xgwm149-Xbarc1118*	6.1-10.8	8.7-22.3	2008,2011,2012,2014,2015
5A	*Xbarc124-Xbarc165*	4.7-6.8	6.2-8.3	2008,2011,2012,2014,2015
6A	*Xbarc232-Xbarc322*	3.2-6.3	5.7-8.9	2011, 2014,2015
6B	*Xbarc134-Xmag4271*	4.1-6.2	6.9-9.3	2011,2012,2015
7A1	*Xbarc222-Xbarc174*	4.2-13.8	8.9-22.6	2008,2011,2012,2014,2015
7A2	*Xwmc405-Xgwm473*	4-7.8	5.9-8.1	2008,2011,2012,2014,2015
7B1	*Xwmc758-Xgwm573*	4.8-24.3	8.3-28.9	2008,2011,2012,2014,2015
7B2	*Xwmc476-Xbarc267*	5.2-12.3	8.6-20.1	2011,2012,2014,2015

Table S11 Grain weight and allele variations of twelve varieties

Accessions	TGW (g)	allele
Baihuomai	23.53	*TaTGW-7Ab*
Waitoubai	26.07	*TaTGW-7Ab*
Zitongnvermai	26.33	*TaTGW-7Ab*
Fulingxuxumai	29.38	*TaTGW-7Ab*
Xinmai18	37.16	*TaTGW-7Ab*
Wangshuibai	37.29	*TaTGW-7Ab*
Zhoumai23	46.98	*TaTGW-7Aa*
Taikong6	47.71	*TaTGW-7Aa*
Xinmai23	48.51	*TaTGW-7Aa*
Wennong6	49.17	*TaTGW-7Aa*
Annong0942	50.09	*TaTGW-7Aa*
Zhengyumai518	51.30	*TaTGW-7Aa*

Table S12 The proportions of two *TaTGW-7A* alleles in RIL (Pop 1), natural population (Pop 2), and Chinese wheat mini-core collections (Pop 3).

Populations	*TaTGW-7Aa*		*TaTGW-7Ab*	
	Number of accessions	Proportion (%)	Number of accessions	Proportion (%)
Pop 1	91	60.67%	59	39.33%
Pop 2	209	85.66%	35	14.34%
Pop 3	167	64.98%	90	35.02%

Table S13 Geographic distribution of *TaTGW-7A* alleles in Pop 3 and Pop 4.

Region[a]	Pop 3		Pop 4	
	TaTGW-7Aa	*TaTGW-7Ab*	*TaTGW-7Aa*	*TaTGW-7Ab*
I	0.39 (9)[b]	0.61 (14)	0.89 (84)	0.11 (10)
II	0.49 (25)	0.51 (24)	0.90 (205)	0.10 (23)
III	0.62 (16)	0.38 (10)	0.88 (53)	0.12 (7)
IV	0.46 (12)	0.54 (16)	0.74 (42)	0.26 (15)
V	0.67 (4)	0.33 (2)	-	-
VI	1.00 (9)	0.00 (0)	77 (20)	23 (6)
VII	0.89 (8)	0.11 (1)	-	-
VIII	0.62 (10)	0.38 (6)	81 (17)	19 (4)
IX	0.86 (6)	0.14 (1)	-	-
X	1.00 (9)	0.00 (0)	67 (10)	33 (5)

[a]I. Northern winter wheat region; II. Yellow and Huai River valley winter wheat region; III. Low and middle Yangtze River valley winter wheat region; IV. Southwestern winter wheat region; V. southern winter wheat region; VI. northeastern spring wheat region; VII. northern spring wheat region; VIII. northwestern spring wheat region; IX. Qinghai-Tibet spring-winter wheat region; X. Xinjiang winter-spring wheat region. [b] The number of wheat varieties.

2. Supplementary Figures

Figure S1 The sequences' alignment between SLAF65386 and 7AS_4248784

```
7AS_4248784.seq                AGCAAACTTCTGGACCTGCAGAGAAATCGGTGGTATATAAATTTTGCCAATAGAAATGTG   60
Hongmangchun21_allele_F.seqRC  ............................................................    0
Hongmangchun21_allele_R.seq    AGCAAACTTCTGGACCTGCAGAGAAATCGGTGGTATATAAATTTTGCCAATAGAAATGTG   60
Jing411_allele_F.seqRC         ............................................................    0
Jing411_allele_R.seq           AGCAAACTTCTGGACCTGCAGAGAAATCGGTGGTATATAAATTTTGCCAATAGAAATGTG   60
Consensus

7AS_4248784.seq                TTCTAAGTAATTTTGCGTGTAGAAGCATCCATGTGAGCAATTTGCAACAAAGAAATTCCT  120
Hongmangchun21_allele_F.seqRC  ............................................................    0
Hongmangchun21_allele_R.seq    TTCTAAGTAATTTTGCG...........................................   77
Jing411_allele_F.seqRC         ............................................................    0
Jing411_allele_R.seq           TTCTAAGTAATTTTGCG...........................................   77
Consensus

7AS_4248784.seq                TTTCATTCAACTAATCAGTCATCAGAATCACATTTAGCGATGCAAGGAAACATGCCTGTT  180
Hongmangchun21_allele_F.seqRC  ............................................................    0
Hongmangchun21_allele_R.seq    ............................................................   77
Jing411_allele_F.seqRC         ............................................................    0
Jing411_allele_R.seq           ............................................................   77
Consensus

7AS_4248784.seq                TGGACCTATCACAAAAGCTCACGAACACCACCCTGCTCCCTCTAACTGGGCACTCTGTCA  240
Hongmangchun21_allele_F.seqRC  ............................................................    0
Hongmangchun21_allele_R.seq    ............................................................   77
Jing411_allele_F.seqRC         ............................................................    0
Jing411_allele_R.seq           ............................................................   77
Consensus

7AS_4248784.seq                CGCATGCAACCTGTGGCCATGAGAAGTTGAGGATAGACACATAGTGCTCCTCCTGTGCCA  300
Hongmangchun21_allele_F.seqRC  ............................................................    0
Hongmangchun21_allele_R.seq    ............................................................   77
Jing411_allele_F.seqRC         ............................................................    0
Jing411_allele_R.seq           ............................................................   77
Consensus

7AS_4248784.seq                TGAAAATAGACCTCAGCGTTGAGTTGTTCGGACAGAAAAGAGAATGTATGACGAAACAGA  360
Hongmangchun21_allele_F.seqRC  ............................................................    0
Hongmangchun21_allele_R.seq    ............................................................   77
Jing411_allele_F.seqRC         ............................................................    0
Jing411_allele_R.seq           ............................................................   77
Consensus

7AS_4248784.seq                AATGAACATTGCGTTCAAGTTCGCAGCGTATCCTCATTTCTCCAACCTCCATAACTTCTA  420
Hongmangchun21_allele_F.seqRC  ...................................................TAACTTCTA    9
Hongmangchun21_allele_R.seq    ............................................................   77
Jing411_allele_F.seqRC         ...................................................TAACTTCTA    9
Jing411_allele_R.seq           ............................................................   77
Consensus

7AS_4248784.seq                TCAAATAATATTAGATCAAGAGTTGTAAAAAAAAATAGCAGTGGGAGAATTTGTGGGCAG  480
Hongmangchun21_allele_F.seqRC  TCAAATAATATTAGATCAAGAGTTGTAAAAAAAAATAGCAGTGGGAGAATTTGTGGGCAG   69
Hongmangchun21_allele_R.seq    ............................................................   77
Jing411_allele_F.seqRC         TCAAATAATATTAGATCAAGAATTGTAAAAAAAAATAGCAGTGGGAGAATTTGTGGGCAG   69
Jing411_allele_R.seq           ............................................................   77
Consensus

7AS_4248784.seq                ATGAGC                                                        486
Hongmangchun21_allele_F.seqRC  ATGAGC                                                         75
Hongmangchun21_allele_R.seq    ......                                                         77
Jing411_allele_F.seqRC         ATGAGC                                                         75
Jing411_allele_R.seq           ......                                                         77
Consensus
```

Figure S2 Sequence alignment between 7AS_4248784 and Traes_7AS_378A12AA9.1

```
7AS_4248784           CCTATCCCTAAAACAGTATCTTATCTTTAAAGACAGATCTATCGTTATACTTGAATACTC   2700
Traes_7AS_378A12AA9.1 ------------------------------------------------------------   2700

7AS_4248784           TTCTTATGCACTGCCCACGAATGTGATCCAAATTTGTGAGATTCGACATGAGTGCGCACA   2760
Traes_7AS_378A12AA9.1 ------------------------------------------------------------   2760

7AS_4248784           GGGTACATGAATGTGATAGAGCATACCGAAATATTGACCACGGCCAAATTACGCAAACGA   2820
Traes_7AS_378A12AA9.1 ------------------------------------------------------------   2820

7AS_4248784           GAAAAAATGGCAAAGCTGGCCGCGAATTTTTCAAGCACAAGAGCATGACCTCAGAAGAGT   2880
Traes_7AS_378A12AA9.1 ------------------------------------------------------------   2880

7AS_4248784           ACAACGGATTGTCACTCTCCATTCTCCACAACCCTCTCAGACCTTTTGGGACCCTTGGAT   2940
Traes_7AS_378A12AA9.1 ------------------------------------------------------------   2940

7AS_4248784           CCTGTCTCTATAAAAGACTATTGAGCTAATCGAACATAGCATCTAATCCTCTGTTTCTTG   3000
Traes_7AS_378A12AA9.1 ------------------------------------------------------------   3000

7AS_4248784           GACCTTGATTTGATTTGCGCAATTGCTTGCATTGAGATCATCTGGCCATCTCCTCAAGAG   3060
Traes_7AS_378A12AA9.1 ------------------------------------------------------------   3060

7AS_4248784           TTTATATAGGCTCCCACTTGTCCTCTTCTAAAGCAAGTAGACTACGTGTCCTAGTAGGAT   3120
Traes_7AS_378A12AA9.1 ------------------------------------------------------------   3120

7AS_4248784           TCCTCGGGGTACCACACCTGTTTGAGTCCTAATAAGATTAGGATTAGAATCGCCATATTC   3180
Traes_7AS_378A12AA9.1 ------------------------------------------------------------   3180

7AS_4248784           AGTTGAGAAAATCCATCTGTTACATGCAAGCTGTAATTATTACCTTACAAGTATGTATTT   3240
Traes_7AS_378A12AA9.1 ------------------------------------------------------------   3240

7AS_4248784           TATGTGCCTTTATTTATGAGGATTTCACATTTCCTAATTTTCTTGCGGTGAGTAAAAGAC   3300
Traes_7AS_378A12AA9.1 ------------------------------------------------------------   3300

7AS_4248784           CATGTAATTCTTTCAGATTTCGTTGATGCGGACGACCGTGGAGGAGAACAAGAAATTTGC   3360
Traes_7AS_378A12AA9.1 ----------------ATTTCGTTGATGCGGACGACCGTGGAGGAGAACAAGAAATTTGC   3360

7AS_4248784           TCAGTTTATTGCTGACAAGCTGAACAAGTCTTTATCTACAGTTACTGTTTGCCTTCCACA   3420
Traes_7AS_378A12AA9.1 TCAGTTTATTGCTGACAAGCTGAACAAGTCTTTATCTACAGTTACTGTTTGCCTTCCACA   3420

7AS_4248784           GAAGGGCATCTCTGCAATTGATGCACCTGGAATGCCGTTTTATGATCCTGAGGCTACATC   3480
Traes_7AS_378A12AA9.1 GAAGGGCATCTCTGCAATTGATGCACCTGGAATGCCGTTTTATGATCCTGAGGCTACATC   3480
```

Figure S2 (continued)

```
7AS_4248784           TGCACTATTGGATGAGTTAAATACTCGTCTTGTCAAAACTGAGAACAGACAGGTTTCTTC   3540
Traes_7AS_378A12AA9.1 TGCACTATTGGATGAGTTAAATACTCGTCTTGTCAAAACTGAGAACAGACAG--------   3540

7AS_4248784           ATGTTACATAATATGTTAGTTGCATCGCTAGAATATGAATAGAAAGGGTCATCGAATGAA   3600
Traes_7AS_378A12AA9.1 ------------------------------------------------------------   3600

7AS_4248784           CTAAGCTGATTGGCTTGTTGACAGCTGAAGCTGCTTCCTTATCATATAAACGATCCTGAA   3660
Traes_7AS_378A12AA9.1 ------------------------CTGAAGCTGCTTCCTTATCATATAAACGATCCTGAA   3660

7AS_4248784           TTTGCCAATGCCTTGGTGGATGCATTCTTGAGTATGGATATAAAGGCCTCTAGTGCCATA   3720
Traes_7AS_378A12AA9.1 TTTGCCAATGCCTTGGTGGATGCATTCTTGAGTATGGATATAAAGGCCTCTAGTGCCATA   3720

7AS_4248784           ACTCAGAAAAACAACATGGTCCTACCAAAGCAAGACACAAATGAAAAGGAATCTTCTTCA   3780
Traes_7AS_378A12AA9.1 ACTCAGAAAAACAACATGGTCCTACCAAAGCAAGACACAAATGAAAAGGAATCTTCTTCA   3780

7AS_4248784           GGACAGAAGACTTCAGATAGTTCTATCATATGGAGACCCCCAGTGGATTTCCCTGATGCA   3840
Traes_7AS_378A12AA9.1 GGACAGAAGACTTCAGATAGTTCTATCATATGGAGACCCCCAGTGGATTTCCCTGATGCA   3840

7AS_4248784           AGACCAGGTTAGCGGTGATTCTGTTCCCATCTTATGCGCCCAAATTTTATTATGCCCACA   3900
Traes_7AS_378A12AA9.1 AGACCAG-----------------------------------------------------   3900

7AS_4248784           CAAACAGACTGTTTATTGAATCACTTGTTTTAGTTTCTTACAAAAAAATCGTGGAAGGTT   3960
Traes_7AS_378A12AA9.1 ------------------------------------------------------------   3960

7AS_4248784           ATCTGGTATGTACCTTGTTATCTGCTATGCCACCTTACAGAAAATCCTTCAGGGTGAAGT   4020
Traes_7AS_378A12AA9.1 ------------------------------------------------------------   4020

7AS_4248784           TCTTATTTCGTTTTTGTTCTCATGCTTCATACCTAGGCTTGTCAGAATTTTTGTGTCTCC   4080
Traes_7AS_378A12AA9.1 ------------------------------------------------------------   4080

7AS_4248784           AATGATCCAAGAGGTCAAGATAGTTGTGAGTGACAACTCCTTTGGGGCTGTGCTTCAAGC   4140
Traes_7AS_378A12AA9.1 ------------------------------------------------------------   4140

7AS_4248784           ATCTGTAGTACGCTTCACCGTTTGATGTTGTCCACCTTTCTCTAATCTTAGTATCATGTA   4200
Traes_7AS_378A12AA9.1 ------------------------------------------------------------   4200

7AS_4248784           ATTATTGTGGGGTTGTCTTGGCGAAGACCGCAAGCACTGGCCCAAGGGCGCACAGAAATG   4260
Traes_7AS_378A12AA9.1 ------------------------------------------------------------   4260

7AS_4248784           GTGCCAGCACTTAGGCACAATTCTGACTTGGGAACAGAAGTCTCTATTCAAAGAGATTTC   4320
Traes_7AS_378A12AA9.1 ------------------------------------------------------------   4320
```

Figure S2 (continued)

```
7AS_4248784            ATTGTGTTCCAAAGGATTCATGTAGTCCTGAAGAAACTCAACGTAGCTGGGGATGAGCTC   4380
Traes_7AS_378A12AA9.1 ------------------------------------------------------------   4380

7AS_4248784            AACATGCACATGGGACAAGCTCGGCGTACATGCCTAACCTTTATGATATCAACCACAAAA   4440
Traes_7AS_378A12AA9.1 ------------------------------------------------------------   4440

7AS_4248784            TACTTGCTATTGTCACCTATTACCTCTCTACTGCGCCAAATCAGAAATGTTCGGTGTCAC   4500
Traes_7AS_378A12AA9.1 ------------------------------------------------------------   4500

7AS_4248784            CTCACGTGTTCGCTGACAAATTAGGATTAACTCCAGCTATTAGTAAGTGCGCTAAGTTTT   4560
Traes_7AS_378A12AA9.1 ------------------------------------------------------------   4560

7AS_4248784            AGTCTATTTGCAGTACAGTATGATTTTACAAAGGGGTAATGTCAAAACTCTAATGATCTT   4620
Traes_7AS_378A12AA9.1 ------------------------------------------------------------   4620

7AS_4248784            ACACATTATAGTACCCGTGTGCACGGATGAAATAACTGTCAAAGGTCAGAAGTTTGGCAT   4680
Traes_7AS_378A12AA9.1 ------------------------------------------------------------   4680

7AS_4248784            TAGATTCTAGGGCATAATAAGCATTCCGAACCCTCACGTGATACCACACAAGTTACATAA   4740
Traes_7AS_378A12AA9.1 ------------------------------------------------------------   4740

7AS_4248784            ATTTCATAATTTTATTTTCAGCTCATTGTCTCCTTGTTTGGCATGACATGTTAAAGGGCG   4800
Traes_7AS_378A12AA9.1 ------------------------------------------------------------   4800

7AS_4248784            TTCGTCATTCATTTCAGTTCTTGAGATCTATTGACACAATTCTATACCCTAATGATTCTA   4860
Traes_7AS_378A12AA9.1 ------------------------------------------------------------   4860

7AS_4248784            ATTGCTCACTCTTCAATGATGACAGAAACTTTGCAAAAAACAAAGTCAATACTACATAAG   4920
Traes_7AS_378A12AA9.1 -------------------------AAACTTTGCAAAAAACAAAGTCAATACTACATAAG   4920

7AS_4248784            TTAAAGCAACAAATCGGTGAGGGTATTCCTGTAATTGGAGCCGGTGCTGGTACGGGCATA   4980
Traes_7AS_378A12AA9.1 TTAAAGCAACAAATCGGTGAGGGTATTCCTGTAATTGGAGCCGGTGCTGGTACGGGCATA   4980

7AS_4248784            TCCGCGAAGTTCGAAGAAGCTGGTGGGGTTGATCTGATTGTGTTGTACAATTCCGGGAGG   5040
Traes_7AS_378A12AA9.1 TCCGCGAAGTTCGAAGAAGCTGGTGGGGTTGATCTGATTGTGTTGTACAATTCCGGGAGG   5040

7AS_4248784            TTTCGTATGGCTGGAAGGGGCTCATTAGCAGGGCTCCTACCATTTGCTGACGCAAATGCA   5100
Traes_7AS_378A12AA9.1 TTTCGTATGGCTGGAAGGGGCTCATTAGCAGGGCTCCTACCATTTGCTGACGCAAATGCA   5100

7AS_4248784            ATTGTACTTGAGATGGCCAATGAAGTGTTGCCTGTAAGTTTTTTTAGTAGTGGGGTTCAT   5160
Traes_7AS_378A12AA9.1 ATTGTACTTGAGATGGCCAATGAAGTGTTGCCTGT-------------------------   5160
```

Figure S2 (continued)

```
7AS_4248784           TCTTTTGTTTTTCTATTATCTGACTTGAGTATAGCTGGAAGTTAATGAGGTGCTAATTTA   5220
Traes_7AS_378A12AA9.1 ------------------------------------------------------------   5220

7AS_4248784           GGTCGTTAAAGAAGTTCCTGTTCTTGCTGGGGTTTGCGCTACTGATCCATTTCGTAGAAT   5280
Traes_7AS_378A12AA9.1 ---CGTTAAAGAAGTTCCTGTTCTTGCTGGGGTTTGCGCTACTGATCCATTTCGTAGAAT   5280

7AS_4248784           GGATTACTTTCTTAAACAGCTAGAAGCCATTGGATTTTGTGGTGTCCAAAATTTTCCTAC   5340
Traes_7AS_378A12AA9.1 GGATTACTTTCTTAAACAGCTAGAAGCCATTGGATTTTGTGGTGTCCAAAATTTTCCTAC   5340

7AS_4248784           GGTTGGTCTGTTTGATGGGAACTTCAGACAGAACTTGGAAGAAACTGGAATGGGCTACAG   5400
Traes_7AS_378A12AA9.1 GGTTGGTCTGTTTGATGGGAACTTCAGACAGAACTTGGAAGAAACTGGAATGGGCTACAG   5400

7AS_4248784           GTATTGATTCACCACATCCTTATTTTTCGGATACATGTGTTATCCATGCCATGGCATGGA   5460
Traes_7AS_378A12AA9.1 ------------------------------------------------------------   5460

7AS_4248784           CATAATATGCATATGAAAAATTCTGGGCTGAATGTAGGATATGGATTCTGTTTCAGTGAC   5520
Traes_7AS_378A12AA9.1 ------------------------------------------------------------   5520

7AS_4248784           TTCACAAATCGTAATGGTTAGTTTCCTTATTTAGTTTTCTGCTGAGGGCAAGATTTGCAT   5580
Traes_7AS_378A12AA9.1 ------------------------------------------------------------   5580

7AS_4248784           GTCTGAATTCTGAAATCTTGCTAAATTCCGCATTTGAGTGTGTGTTCAAAGAAATTATGT   5640
Traes_7AS_378A12AA9.1 ------------------------------------------------------------   5640

7AS_4248784           TAAATTGATCTTGGTCTTTTGCCAGCATGGAAGTGGAGATGATCTCAAGGGCTCACAGCA   5700
Traes_7AS_378A12AA9.1 -------------------------CATGGAAGTGGAGATGATCTCAAGGGCTCACAGCA   5700

7AS_4248784           TGGGTTTCCTGACGACCCCGTATGCTTTCAATCCAGAAGAAGGCGCTGCCATGGCCAAGG   5760
Traes_7AS_378A12AA9.1 TGGGTTTCCTGACGACCCCGTATGCTTTCAATCCAGAAGAAGGCGCTGCCATGGCCAAGG   5760

7AS_4248784           CCGGAGCGCACATTGTAGTCGCGCATATGGGCCTCACAACAGCTGGATCGATCGGCGCAA   5820
Traes_7AS_378A12AA9.1 CCGGAGCGCACATTGTAGTCGCGCATATGGGCCTCACAACAGCTGGATCGATCGGCGCAA   5820

7AS_4248784           AGACGGCCGCCACATTAGATGACAGCATTGTCCGGGTTCAAGCCATTGCCGATGCCGCGG   5880
Traes_7AS_378A12AA9.1 AGACGGCCGCCACATTAGATGACAGCATTGTCCGGGTTCAAGCCATTGCCGATGCCGCGG   5880

7AS_4248784           TCGGCGTCAACCCTGACATCATCGTTCTCTGCCATGGAGGTAAGCAGTCTCTGCTTCATG   5940
Traes_7AS_378A12AA9.1 TCGGCGTCAACCCTGACATCATCGTTCTCTGCCATGGAGGTAAGCAGTCTCTGCTTCATG   5940

7AS_4248784           CAGACCTCAAGTAACAGATATGGTGTCTTGATAAATCATGTGATTCTGATGCACAATTCC   6000
Traes_7AS_378A12AA9.1 ------------------------------------------------------------   6000
```

Figure S2 (continued)

```
7AS_4248784             ACCATCAGGTCCCATATCAGGGCCCCGAGAGGCGGAGTTTGTCCTGAAGAACACGAACCG   6060
Traes_7AS_378A12AA9.1   ----------CCCATATCAGGGCCCCGAGAGGCGGAGTTTGTCCTGAAGAACACGAACCG   6060

7AS_4248784             GGTCCATGGATTCTACGGCGCCTCGAGCATGGAGAGGCTGCCGGTTGAGCAGGCCATCAC   6120
Traes_7AS_378A12AA9.1   GGTCCATGGATTCTACGGCGCCTCGAGCATGGAGAGGCTGCCGGTTGAGCAGGCCATCAC   6120

7AS_4248784             AAACACCATGAGGGAGTACAAACGCATGTCTCTGAAATGAGGTTCGGTGGCTTTACCAGG   6180
Traes_7AS_378A12AA9.1   AAACACCATGAGGGAGTACAAACGCATGTCTCTGAAATGA--------------------   6180

7AS_4248784             TGGGCGGATGAAGCGCGCCCGGCGTTCGTCAGGCTGTTGTCACATGTAAATTGTTACTGA   6240
Traes_7AS_378A12AA9.1   ------------------------------------------------------------   6240

7AS_4248784             AGGTGCCTTGTGCGTTTGAACATATAATAAAAGATACCGACGAAACTATAGGGTGTGTGA   6300
Traes_7AS_378A12AA9.1   ------------------------------------------------------------   6300

7AS_4248784             CGTGGCATGCCGTCTCGTTGGTCCAGGCGGTCATGCATTACAGGATGCCCATCAATGAGA   6360
Traes_7AS_378A12AA9.1   ------------------------------------------------------------   6360

7AS_4248784             TATGATCTTTTCCAGGCGGTCATACATTACAGGACGCCCATCAATGTTTTTCACTAACCC   6420
Traes_7AS_378A12AA9.1   ------------------------------------------------------------   6420
```

Figure S3 QTL mapping for TGW in the RIL population derived from the cross of Jing 411 × Hongmangchun 21. Logarithm of odds (LOD) contours are obtained by composite interval mapping; LOD thresholds = 2.5. Labels W1, W2, W3, W4 and W5 refer to TGW in 2008, 2011, 2012, 2014 and 2015, respectively.

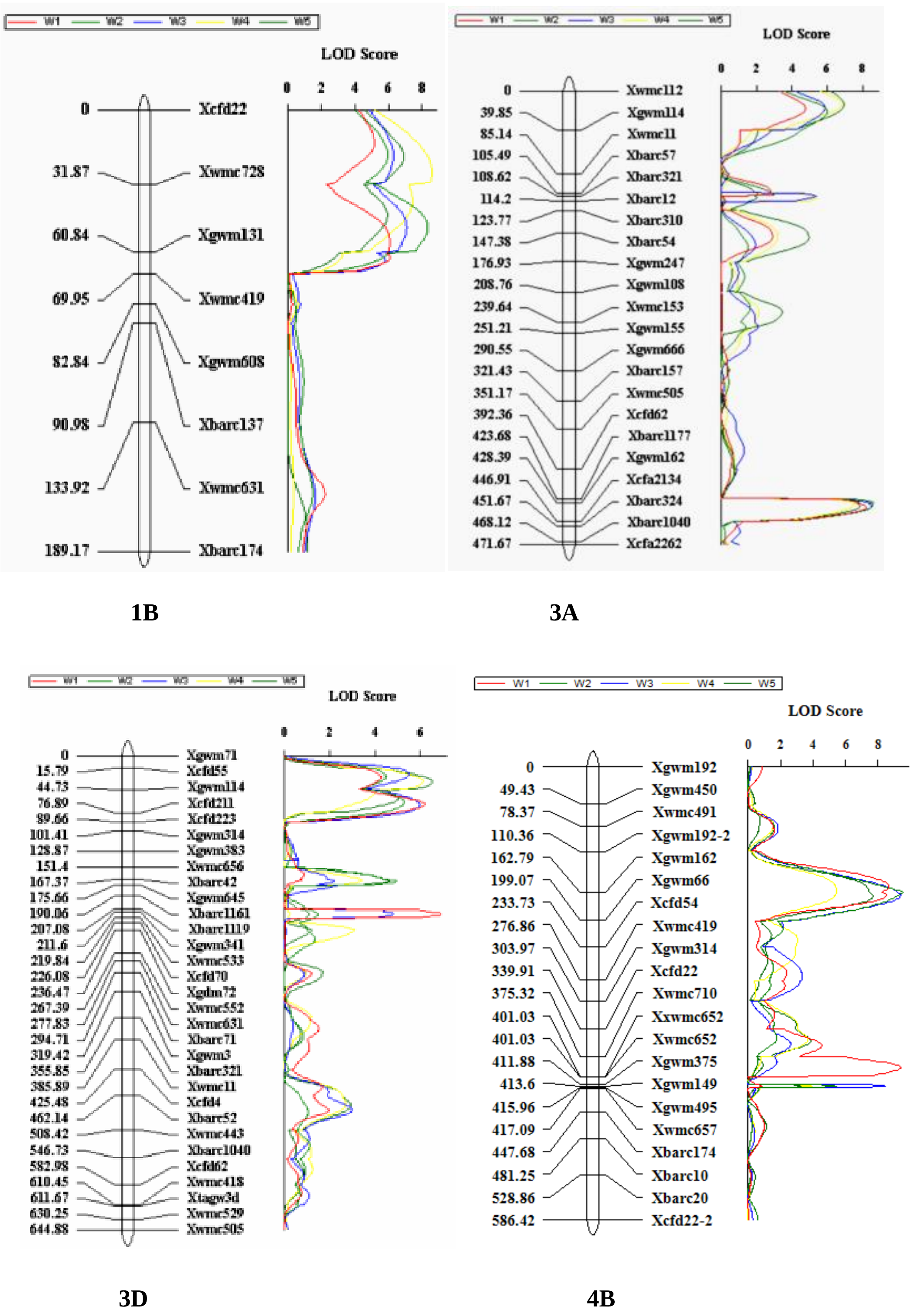

Figure S3 (continued)

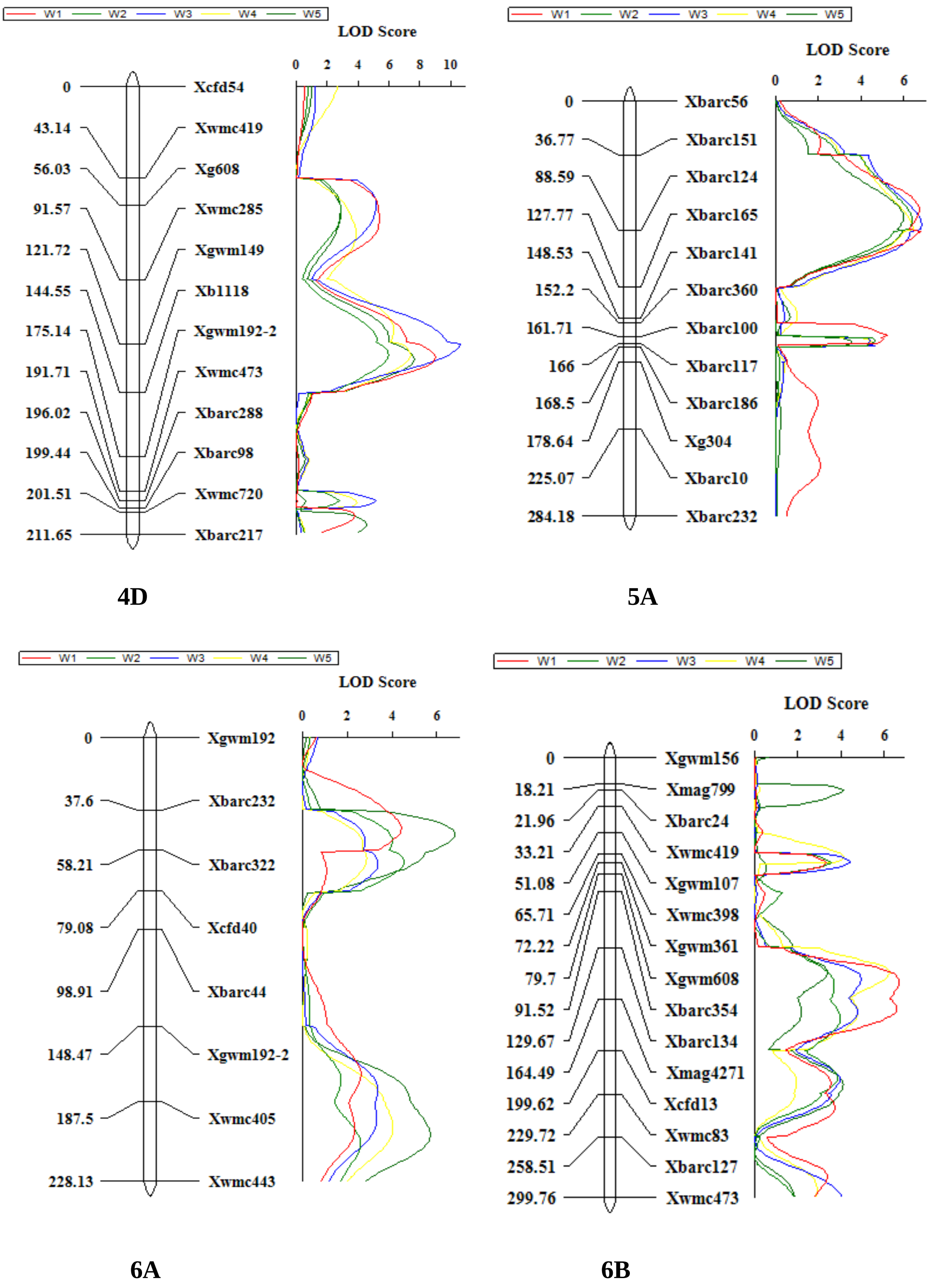

Figure S3 (continued)

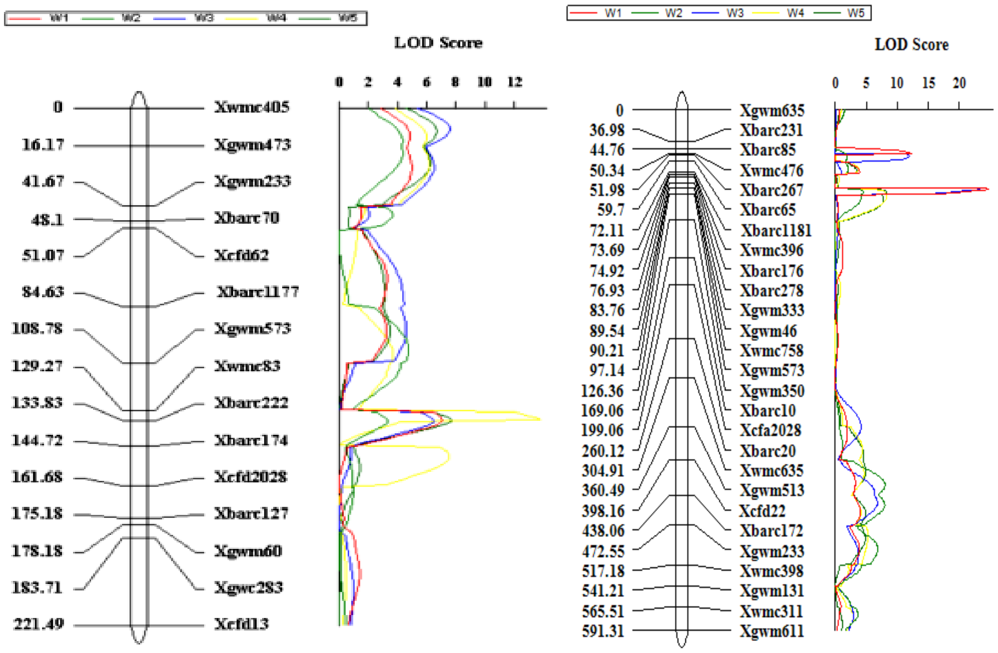

7A **7B**

Figure S4 Sequence alignment between *TaTGW-7Aa* and *TaTGW-7Ab*. The red box means SNP. The *TaTGW-7Aa* allele is corresponding to higher TGW and *TaTGW-7Ab* is to lower TGW.

```
TaTGW-7Aa ATCGCCAGCCGTGCAGATCGGAGAATCGCAATCCGATCCCAAATCAGGCCCCAGCACCAG   60
TaTGW-7Ab ATCGCCAGCCGTGCAGATCGGAGAATCGCAATCCGATCCCAAATCAGGCCCCAGCACCAG   60

TaTGW-7Aa CGTGCCCAACGTGGCGCCGGCAGCGGGGCACACGACACCGCTATGGAAATGGAATGCAAG  120
TaTGW-7Ab CGTGCCCAACGTGGCGCCGGCAGCGGGGCACACGACACCGCTATGGAAATGGAATGCAAG  120

TaTGW-7Aa TCAACACACCTCACTTCCACTGTTTGCCCGTCCCCACTACCAGCCTAGCTACATACACGC  180
TaTGW-7Ab TCAACACACCTCACTTCCACTGTTTGCCCGTCCCCACTACCAGCCTAGCTACATACACGC  180

TaTGW-7Aa ACCAACCCATGGGCCATGATCCACCCATCCCTCCATCCGTCGCGGTTCCCCCTGCTGAGC  240
TaTGW-7Ab ACCAACCCATGGGCCATGATCCACCCATCCCTCCATCCGTCGCGGTTCCCCCTGCTGAGC  240

TaTGW-7Aa TCCCGATCTCTGCTCCGAGGCGTCGGCAATGGAGGTGCTCTGCATCGGGACGGCCGACAC  300
TaTGW-7Ab TCCCGATCTCTGCTCCGAGGCGTCGGCAATGGAGGTGCTCTGCATCGGGACGGCCGACAC  300

TaTGW-7Aa CAAGCTGGAGGAGCTGCTCTTCCTCGCCACTCGCCTCCGCTCCAGCCTCGCCGCCTCCGT  360
TaTGW-7Ab CAAGCTGGAGGAGCTGCTCTTCCTCGCCACTCGCCTCCGCTCCAGCCTCGCCGCCTCCGC  360

TaTGW-7Aa CTCCGCCTCCGCCCCAAAGGTACTGCTACACTTACACTGTCCGCCTATCTTCTCGTCCCC  420
TaTGW-7Ab CTCCGCCTCCGCCCCAAAGGTACTGCTACACTTACACTGTCCGCCTATCTTCTCGTCCCC  420

TaTGW-7Aa GCCTGCAACTAGGTGAGGAGTTCAGCTATCCATTCGCATGCTTGAAGCGATCGTCCAGAA  480
TaTGW-7Ab GCCTGCAACTAGGTGAGGAGTTCAGCTATCCATTCGCATGCTTGAAGCGATCGTCCAGAA  480

TaTGW-7Aa TGGGGTGAACACGCATTTGGCGTCACCTACTTTAGAAACTACTATAACTACAGTGCAGTT  540
TaTGW-7Ab TGGGGTGAACACGCATTTGGCGTCACCTACTTTAGAAACTACTATAACTACAGTGCAGTT  540

TaTGW-7Aa TTGAGGGAGTATTCCTTCACTGAACGCAGAAACCGGAGTTCCTTCTCTGAACGTGCAAAC  600
TaTGW-7Ab TTGAGGGAGTATTCCTTCACTGAACGCAGAAACCGGAGTTCCTTCTCTGAACGTGCAAAC  600

TaTGW-7Aa TGCGTCATAGCATTGGGAACTACAGTAGCAGGGTAACATATGGATTATGCTATTTCTCAT  660
TaTGW-7Ab TGCGTCATAGCATTGGGAACTACAGTAGCAGGGTAACATATGGATTATGCTATTTCTCAT  660

TaTGW-7Aa CTTGTGTTCTTGGAACTAGTTATTATATTTCCGGTTGGTAATTCTGGGATTGCCATATCA  720
TaTGW-7Ab CTTGTGTTCTTGGAACTAGTTATTATATTTCCGGTTGGTAATTCTGGGATTGCCATATCA  720

TaTGW-7Aa TAAAAAGAGCGATGCTTCTAGATTTGAAGTTTAACGAAATCATGTGATCATGCGCTCTTC  780
TaTGW-7Ab TAAAAAGAGCGATGCTTCTAGATTTGAAGTTTAACGAAATCATGTGATCATGCGCTCTTC  780
```

Figure S4 (continued)

TaTGW-7Aa CGGTTTAGCAGTTGTGTTGCGTTTTTAGCCTATGGAAATTCCCTGTGGCTGCTTATTGAA 840

TaTGW-7Ab CGGTTTAGCAGTTGTGTTGCGTTTTTAGCCTATGGAAATTCCCTGTGGCTGCTTATTGAA 840

TaTGW-7Aa CAGGTGACAACAGATGCCATGTCTAATGTTTTGCTCAAGGAGTTCTTATGCAACTTTCAC 900

TaTGW-7Ab CAGGTGACAACAGATGCCATGTCTAATGTTTTGCTCAAGGAGTTCTTATGCAACTTTCAC 900

TaTGW-7Aa CAAAGTACATTTCCTGCTTAAGGCTCAGAGTACATGAGTCCTTCATTAATTTGTGTTATC 960

TaTGW-7Ab CAAAGTACATTTCCTGCTTAAGGCTCAGAGTACATGAGTCCTTCATTAATTTGTGTTATC 960

TaTGW-7Aa CACATACAATAGCCTTAAATATCGTTCGTCTACTCTCAGTAATAGACAATCATTCGCCAA 1020

TaTGW-7Ab CACATACAATAGCCTTAAATATCGaTCGTCTACTCTCAGTAATAGACAATCATTCGCCAA 1020

TaTGW-7Aa GTATCAACAATTGCTACTCCCTACGTTCCATAATTCTTGTCGTGGTTAAACATTGGAGCA 1080

TaTGW-7Ab GTATCAACAATTGCTACTCCCTACGTTCCATAATTCTTGTCGTGGTTAAACATTGGAGCA 1080

TaTGW-7Aa TCCAAACTGCTGAAGCAGGACACATCTTTATGTGCACCTGTGTGTAGTGCACCCTTGGAG 1140

TaTGW-7Ab TCCAAACTGCTGAAGCAGGACACATCTTTATGTGCACCTGTGTGTAGTGCACCCTTGGAG 1140

TaTGW-7Aa TTGCCCTGTAGGTTTATTATTTGTTTTGTTAACTCGAGCATAACATGTGGTCTATTGAAA 1200

TaTGW-7Ab TTGCCCTGTAGGTTTATTATTTGTTTTGTTAACTCGAGCATAACATGTGGTCTATTGAAA 1200

TaTGW-7Aa TGGTAGGTTAAAGTAAGCATAGTGGATGTCTCCACAACTAAAACAGTACCAACACAAGAT 1260

TaTGW-7Ab TGGTAGGTTAAAGTAAGCATAGTGGATGTCTCCACAACTAAAACAGTACCAACACAAGAT 1260

TaTGW-7Aa TCTAAAGATATTGCAGTTATTGCAAGAGATACAGTTCTCTCATGCCATCCGGATTCCAGC 1320

TaTGW-7Ab TCTAAAGATATTGCAGTTATTGCAAGAGATACAGTTCTCTCATGCCATCCGGATTCCAGC 1320

TaTGW-7Aa CAGCAAGATCTTCCAGATGACAGAGGTGAAGCGATTGCGCTTATGTCAAAGGCCCTTCAG 1380

TaTGW-7Ab CAGCAAGATCTTCCAGATGACAGAGGTGAAGCGATTGCGCTTATGTCAAAGGCCCTTCAG 1380

TaTGW-7Aa AGCTTTCTGAAAAACAGATATGAGGCCGGCACCCTGGTTGCTGCTGTTGGCCTAGGAGGA 1440

TaTGW-7Ab AGCTTTCTGAAAAACAGATATGAGGCCGGCACCCTGGTTGCTGCTGTTGGCCTAGGAGGA 1440

TaTGW-7Aa AGTGGAGGAACCGCACTAATTGCCCCTGCTCTAAGATCCCTACCACTTGGAGTGCCTAAG 1500

TaTGW-7Ab AGTGGAGGAACCGCACTAATTGCCCCTGCTCTAAGATCCCTACCACTTGGAGTGCCTAAG 1500

TaTGW-7Aa CTTATTGTATCCACTGTTGCTAGTGGCAATACTGCACCCTATGTTGGAACATCTGACTTG 1560

TaTGW-7Ab CTTATTGTATCCACTGTTGCTAGTGGCAATACTGCACCCTATGTTGGAACATCTGACTTG 1560

TaTGW-7Aa GTATTGTTTCCTTCAGTTGTTGACATATGTGGAATAAACAGTGTCAGCCGTGTTATATTG 1620

TaTGW-7Ab GTATTGTTTCCTTCAGTTGTTGACATATGTGGAATAAACAGTGTCAGCCGTGTTATATTG 1620

Figure S4 (continued)

TaTGW-7Aa TCGAATGCTGCTTCAGCTGTTGCCGGATTGGTATGTGGGATATTAATGGCTTCCAGTGAA 1680

TaTGW-7Ab TCGAATGCTGCTTCAGCTGTTGCCGGATTGGTATGTGGGATATTAATGGCTTCCAGTGAA 1680

TaTGW-7Aa TCAGATGAAACAGACACAAAGCTGACTGTTGGAATTACAATGTTTGGTGTTACCACACAA 1740

TaTGW-7Ab TCAGATGAAACAGACACAAAGCTGACTGTTGGAATTACAATGTTTGGTGTTACCACACAA 1740

TaTGW-7Aa TGTGCAAATGCGGTCAAAGATAGACTGAACAAAGAAGGGTATGAGACGCTTGTATTCCAT 1800

TaTGW-7Ab TGTGCAAATGCGGTCAAAGATAGACTGAACAAAGAAGGGTATGAGACGCTTGTATTCCAT 1800

TaTGW-7Aa GCCACTGGTGTCGGAGGCAAAGCAATGGAAGAACTAGTTAGAGGTGGTTTCATACAGGTA 1860

TaTGW-7Ab GCCACTGGTGTCGGAGGCAAAGCAATGGAAGAACTAGTTAGAGGTGGTTTCATACAGGTA 1860

TaTGW-7Aa ATTAATACTTTCATGTGGTTTTGTACGGTTTTGCCCTTGTCTATTTTTCTATAAGATTTT 1920

TaTGW-7Ab ATTAATACTTTCATGTGGTTTTGTACGGTTTTGCCCTTGTCTATTTTTCTATAAGATTTT 1920

TaTGW-7Aa GTTCTTGTGGAAACTATGTTTTTCAGTTCCTGAACTCTGATGGTAAACTCATTGCCAGAC 1980

TaTGW-7Ab GTTCTTGTGGAAACTATGTTTTTCAGTTCCTGAACTCTGATGGTAAACTCATTGCCAGAC 1980

TaTGW-7Aa AGAAACTCCACTGGTGTTAATTTACTGTCTGTAGAGTTTTTCATCTTACTTCCATTTGGT 2040

TaTGW-7Ab AGAAACTCCACTGGTGTTAATTTACTGTCTGTAGAGTTTTTCATCTTACTTCCATTTGGT 2040

TaTGW-7Aa TGCATGTAGGGTGTATTGGACATAACAACAACAGAAGTTGCGGATTACATTGTTGGAGGT 2100

TaTGW-7Ab TGCATGTAGGGTGTATTGGACATAACAACAACAGAAGTTGCGGATTACATTGTTGGAGGT 2100

TaTGW-7Aa ATCATGGCATGTGATGAGACCAGGTTTGATGCGGTTATAGATAAAAAGATTCCTCTGGTT 2160

TaTGW-7Ab ATCATGGCATGTGATGAGACCAGGTTTGATGCGGTTATAGATAAAAAGATTCCTCTGGTT 2160

TaTGW-7Aa CTCAGTGTTGGGGCCTTGGATATGGTTAACTTTGGAGCTCATGATACAATACCTGCTGCT 2220

TaTGW-7Ab CTCAGTGTTGGGGCCTTGGATATGGTTAACTTTGGAGCTCATGATACAATACCTGCTGCT 2220

TaTGW-7Aa TTCTCAGACAGAAAGATCCACATACATAATGAACAGGTTACACATAGCATATTTTCCTAT 2280

TaTGW-7Ab TTCTCAGACAGAAAGATCCACATACATAATGAACAGGTTACACATAGCATATTTTCCTAT 2280

TaTGW-7Aa TTTGGACCTTGTTTTGCTTCCTAAATTTACTTGGTCTTTGCCAAATTTTATTTATGATGT 2340

TaTGW-7Ab TTTGGACCTTGTTTTGCTTCCTAAATTTACTTGGTCTTTGCCAAATTTTATTTATGATGT 2340

TaTGW-7Aa ATATATTTGCTATGAATACTGAATCATAAATATATTGTTAGGTTAGCACAGTGATATGTT 2400

TaTGW-7Ab ATATATTTGCTATGAATACTGAATCATAAATATATTGTTAGGTTAGCACAGTGATATGTT 2400

TaTGW-7Aa TACCAGATTAATATGCTAGGATATCATTACAGACAATGCTATTATGGTACCAAGAGTAAG 2460

TaTGW-7Ab TACCAGATTAATATGCTAGGATATCATTACAGACAATGCTATTATGGTACCAAGAGTAAG 2460

Figure S4 (continued)

```
TaTGW-7Aa GTTGTGGATATATGTACATAGATTGGTAGATATATAAGAGAGGGGTAGGATGAAGGAAGA   2520
TaTGW-7Ab GTTGTGGATATATGTACATAGATTGGTAGATATATAAGAGAGGGGTAGGATGAAGGAAGA   2520

TaTGW-7Aa ATTCACATTTTTTAAAAAACAGGTGTCTCTAACAACACGTGTCCTTCTGAACAAGAGTCT   2580
TaTGW-7Ab ATTCACATTTTTaAAAAAACAGGTGTCTCTAACAACACGTGTCCTTCTGAACAAGAGTCT   2580

TaTGW-7Aa CTAACAAGTAAATCCATAGACGAGACATAGTCTAGTCAAACACTATTTCTAACAACCCAA   2640
TaTGW-7Ab CTAACAAGTAAATCCATAGACGAGACATAGTCTAGTCAAACACTATTTCTAACAACCCAA   2640

TaTGW-7Aa CCTATCCCTAAAACAGTATCTTATCTTTAAAGACAGATCTATCGTTATACTTGAATACTC   2700
TaTGW-7Ab CCTATCCCTAAAACAGTATCTTATCTTTAAAGACAGATCTATCGTTATACTTGAATACTC   2700

TaTGW-7Aa TTCTTATGCACTGCCCACGAATGTGATCCAAATTTGTGAGATTCGACATGAGTGCGCACA   2760
TaTGW-7Ab TTCTTATGCACTGCCCACGAATGTGATCCAAATTTGTGAGATTCGACATGAGTGCGCACA   2760

TaTGW-7Aa GGGTACATGAATGTGATAGAGCATACCGAAATATTGACCACGGCCAAATTACGCAAACGA   2820
TaTGW-7Ab GGGTACATGAATGTGATAGAGCATACCGAAATATTGACCACGGCCAAATTACGCAAACGA   2820

TaTGW-7Aa GAAAAAATGGCAAAGCTGGCCGCGAATTTTTCAAGCACAAGAGCATGACCTCAGAAGAGT   2880
TaTGW-7Ab GAAAAAATGGCAAAGCTGGCCGCGAATTTTTCAAGCACAAGAGCATGACCTCAGAAGAGT   2880

TaTGW-7Aa ACAACGGATTGTCACTCTCCATTCTCCACAACCCTCTCAGACCTTTTGGGACCCTTGGAT   2940
TaTGW-7Ab ACAACGGATTGTCACTCTCCATTCTCCACAACCCTCTCAGACCTTTTGGGACCCTTGGAT   2940

TaTGW-7Aa CCTGTCTCTATAAAAGACTATTGAGCTAATCGAACATAGCATCTAATCCTCTGTTTCTTG   3000
TaTGW-7Ab CCTGTCTCTATAAAAGACTATTGAGCTAATCGAACATAGCATCTAATCCTCTGTTTCTTG   3000

TaTGW-7Aa GACCTTGATTTGATTTGCGCAATTGCTTGCATTGAGATCATCTGGCCATCTCCTCAAGAG   3060
TaTGW-7Ab GACCTTGATTTGATTTGCGCAATTGCTTGCATTGAGATCATCTGGCCATCTCCTCAAGAG   3060

TaTGW-7Aa TTTATATAGGCTCCCACTTGTCCTCTTCTAAAGCAAGTAGACTACGTGTCCTAGTAGGAT   3120
TaTGW-7Ab TTTATATAGGCTCCCACTTGTCCTCTTCTAAAGCAAGTAGACTACGTGTCCTAGTAGGAT   3120

TaTGW-7Aa TCCTCGGGGTACCACACCTGTTTGAGTCCTAATAAGATTAGGATTAGAATCGCCATATTC   3180
TaTGW-7Ab TCCTCGGGGTACCACACCTGTTTGAGTCCTAATAAGATTAGGATTAGAATCGCCATATTC   3180

TaTGW-7Aa AGTTGAGAAAATCCATCTGTTACATGCAAGCTGTAATTATTACCTTACAAGTATGTATTT   3240
TaTGW-7Ab AGTTGAGAAAATCCATCTGTTACATGCAAGCTGTAATTATTACCTTACAAGTATGTATTT   3240

TaTGW-7Aa TATGTGCCTTTATTTATGAGGATTTCACATTTCCTAATTTTCTTGCGGTGAGTAAAAGAC   3300
TaTGW-7Ab TATGTGCCTTTATTTATGAGGATTTCACATTTCCTAATTTTCTTGCGGTGAGTAAAAGAC   3300
```

Figure S4 (continued)

```
TaTGW-7Aa CATGTAATTCTTTCAGATTTCGTTGATGCGGACGACCGTGGAGGAGAACAAGAAATTTGC   3360
TaTGW-7Ab CATGTAATTCTTTCAGATTTCGTTGATGCGGACGACCGTGGAGGAGAACAAGAAATTTGC   3360
Consensus    catgtaattctttcagatttcgttgatgcggacgaccgtggaggagaacaagaaatttgc

TaTGW-7Aa TCAGTTTATTGCTGACAAGCTGAACAAGTCTTTATCTACAGTTACTGTTTGCCTTCCACA   3420
TaTGW-7Ab TCAGTTTATTGCTGACAAGCTGAACAAGTCTTTATCTACAGTTACTGTTTGCCTTCCACA   3420

TaTGW-7Aa GAAGGGCATCTCTGCAATTGATGCACCTGGAATGCCGTTTTATGATCCTGAGGCTACATC   3480
TaTGW-7Ab GAAGGGCATCTCTGCAATTGATGCACCTGGAATGCCGTTTTATGATCCTGAGGCTACATC   3480

TaTGW-7Aa TGCACTATTGGATGAGTTAAATACTCGTCTTGTCAAAACTGAGAACAGACAGGTTTCTTC   3540
TaTGW-7Ab TGCACTATTGGATGAGTTAAATACTCGTCTTGTCAAAACTGAGAACAGACAGGTTTCTTC   3540

TaTGW-7Aa ATGTTACATAATATGTTAGTTGCATCGCTAGAATATGAATAGAAAGGGTCATCGAATGAA   3600
TaTGW-7Ab ATGTTACATAATATGTTAGTTGCATCGCTAGAATATGAATAGAAAGGGTCATCGAATGAA   3600

TaTGW-7Aa CTAAGCTGATTGGCTTGTTGACAGCTGAAGCTGCTTCCTTATCATATAAACGATCCTGAA   3660
TaTGW-7Ab CTAAGCTGATTGGCTTGTTGACAGCTGAAGCTGCTTCCTTATCATATAAACGATCCTGAA   3660

TaTGW-7Aa TTTGCCAATGCCTTGGTGGATGCATTCTTGAGTATGGATATAAAGGCCTCTAGTGCCATA   3720
TaTGW-7Ab TTTGCCAATGCCTTGGTGGATGCATTCTTGAGTATGGATATAAAGGCCTCTAGTGCCATA   3720

TaTGW-7Aa ACTCAGAAAAACAACATGGTCCTACCAAAGCAAGACACAAATGAAAAGGAATCTTCTTCA   3780
TaTGW-7Ab ACTCAGAAAAACAACATGGTCCTACCAAAGCAAGACACAAATGAAAAGGAATCTTCTTCA   3780

TaTGW-7Aa GGACAGAAGACTTCAGATAGTTCTATCATATGGAGACCCCCAGTGGATTTCCCTGATGCA   3840
TaTGW-7Ab GGACAGAAGACTTCAGATAGTTCTATCATATGGAGACCCCCAGTGGATTTCCCTGATGCA   3840

TaTGW-7Aa AGACCAGGTTAGCGGTGATTCTGTTCCCATCTTATGCGCCCAAATTTTATTATGCCCACA   3900
TaTGW-7Ab AGACCAGGTTAGCGGTGATTCTGTTCCCATCTTATGCGCCCAAATTTTATTATGCCCACA   3900

TaTGW-7Aa CAAACAGACTGTTTATTGAATCACTTGTTTTAGTTTCTTACAAAAAAATCGTGGAAGGTT   3960
TaTGW-7Ab CAAACAGACTGTTTATTGAATCACTTGTTTTAGTTTCTTACAAAAAAATCGTGGAAGGTT   3960

TaTGW-7Aa ATCTGGTATGTACCTTGTTATCTGCTATGCCACCTTACAGAAAATCCTTCAGGGTGAAGT   4020
TaTGW-7Ab ATCTGGTATGTACCTTGTTATCTGCTATGCCACCTTACAGAAAATCCTTCAGGGTGAAGT   4020

TaTGW-7Aa TCTTATTTCGTTTTTGTTCTCATGCTTCATACCTAGGCTTGTCAGAATTTTTGTGTCTCC   4080
TaTGW-7Ab TCTTATTTCGTTTTTGTTCTCATGCTTCATACCTAGGCTTGTCAGAATTTTTGTGTCTCC   4080

TaTGW-7Aa AATGATCCAAGAGGTCAAGATAGTTGTGAGTGACAACTCCTTTGGGGCTGTGCTTCAAGC   4140
TaTGW-7Ab AATGATCCAAGAGGTCAAGATAGTTGTGAGTGACAACTCCTTTGGGGCTGTGCTTCAAGC   4140
```

Figure S4 (continued)

TaTGW-7Aa ATCTGTAGTACGCTTCACCGTTTGATGTTGTCCACCTTTCTCTAATCTTAGTATCATGTA 4200
TaTGW-7Ab ATCTGTAGTACGCTTCACCGTTTGATGTTGTCCACCTTTCTCTAATCTTAGTATCATGTA 4200

TaTGW-7Aa ATTATTGTGGGGTTGTCTTGGCGAAGACCGCAAGCACTGGCCCAAGGGCGCACAGAAATG 4260
TaTGW-7Ab ATTATTGTGGGGTTGTCTTGGCGAAGACCGCAAGCACTGGCCCAAGGGCGCACAGAAATG 4260

TaTGW-7Aa GTGCCAGCACTTAGGCACAATTCTGACTTGGGAACAGAAGTCTCTATTCAAAGAGATTTC 4320
TaTGW-7Ab GTGCCAGCACTTAGGCACAATTCTGACTTGGGAACAGAAGTCTCTATTCAAAGAGATTTC 4320

TaTGW-7Aa ATTGTGTTCCAAAGGATTCATGTAGTCCTGAAGAAACTCAACGTAGCTGGGGATGAGCTC 4380
TaTGW-7Ab ATTGTGTTCCAAAGGATTCATGTAGTCCTGAAGAAACTCAACGTAGCTGGGGATGAGCTC 4380

TaTGW-7Aa AACATGCACATGGGACAAGCTCGGCGTACATGCCTAACCTTTATGATATCAACCACAAAA 4440
TaTGW-7Ab AACATGCACATGGGACAAGCTCGGCGTACATGCCTAACCTTTATGATATCAACCACAAAA 4440

TaTGW-7Aa TACTTGCTATTGTCACCTATTACCTCTCTACTGCGCCAAATCAGAAATGTTCGGTGTCAC 4500
TaTGW-7Ab TACTTGCTATTGTCACCTATTACCTCTCTACTGCGCCAAATCAGAAATGTTCGGTGTCAC 4500

TaTGW-7Aa CTCACGTGTTCGCTGACAAATTAGGATTAACTCCAGCTATTAGTAAGTGCGCTAAGTTTT 4560
TaTGW-7Ab CTCACGTGTTCGCTGACAAATTAGGATTAACTCCAGCTATTAGTAAGTGCGCTAAGTTTT 4560

TaTGW-7Aa AGTCTATTTGCAGTACAGTATGATTTTACAAAGGGGTAATGTCAAAACTCTAATGATCTT 4620
TaTGW-7Ab AGTCTATTTGCAGTACAGTATGATTTTACAAAGGGGTAATGTCAAAACTCTAATGATCTT 4620

TaTGW-7Aa ACACATTATAGTACCCGTGTGCACGGATGAAATAACTGTCAAAGGTCAGAAGTTTGGCAT 4680
TaTGW-7Ab ACACATTATAGTACCCGTGTGCAtGGATGAAATAACTGTCAAAGGTCAGAAGTTTGGCAT 4680

TaTGW-7Aa TAGATTCTAGGGCATAATAAGCATTCCGAACCCTCACGTGATACCACACAAGTTACATAA 4740
TaTGW-7Ab TAGATTCTAGGGCATAATAAGCATTCCGAACCCTCACGTGATACCACACAAGTTACATAA 4740

TaTGW-7Aa ATTTCATAATTTTATTTTCAGCTCATTGTCTCCTTGTTTGGCATGACATGTTAAAGGGCG 4800
TaTGW-7Ab ATTTCATAATTTTATTTTCAGCTCATTGTCTCCTTGTTTGGCATGACATGTTAAAGGGCG 4800

TaTGW-7Aa TTCGTCATTCATTTCAGTTCTTGAGATCTATTGACACAATTCTATACCCTAATGATTCTA 4860
TaTGW-7Ab TTCGTCATTCATTTCAGTTCTTGAGATCTATTGACACAATTCTATACCCTAATGATTCTA 4860

TaTGW-7Aa ATTGCTCACTCTTCAATGATGACAGAAACTTTGCAAAAAACAAAGTCAATACTACATAAG 4920
TaTGW-7Ab ATTGCTCACTCTTCAATGATGACAGAAACTTTGCAAAAAACAAAGTCAATACTACATAAG 4920

TaTGW-7Aa TTAAAGCAACAAATCGGTGAGGGTATTCCTGTAATTGGAGCCGGTGCTGGTACGGGCATA 4980
TaTGW-7Ab TTAAAGCAACAAATCGGTGAGGGTATTCCTGTAATTGGAGCCGGTGCTGGTACGGGCATA 4980

Figure S4 (continued)

TaTGW-7Aa TCCGCGAAGTTCGAAGAAGCTGGTGGGGTTGATCTGATTGTGTTGTACAATTCCGGGAGG 5040

TaTGW-7Ab TCCGCGAAGTTCGAAGAAGCTGGTGGGGTTGATCTGATTGTGTTGTACAATTCCGGGAGG 5040

TaTGW-7Aa TTTCGTATGGCTGGAAGGGGCTCATTAGCAGGGCTCCTACCATTTGCTGACGCAAATGCA 5100

TaTGW-7Ab TTTCGTATGGCTGGAAGGGGCTCATTAGCAGGGCTCCTACCATTTGCTGACGCAAATGCA 5100

TaTGW-7Aa ATTGTACTTGAGATGGCCAATGAAGTGTTGCCTGTAAGTTTTTTAGTAGTGGGGTTCAT 5160

TaTGW-7Ab ATTGTACTTGAGATGGCCAATGAAGTGTTGCCTGTAAGTTTTTTAGTAGTGGGGTTCAT 5160

TaTGW-7Aa TCTTTTGTTTTTCTATTATCTGACTTGAGTATAGCTGGAAGTTAATGAGGTGCTAATTTA 5220

TaTGW-7Ab TCTTTTGTTTTTCTATTATCTGACTTGAGTATAGCTGGAAGTTAATGAGGTGCTAATTTA 5220

TaTGW-7Aa GGTCGTTAAAGAAGTTCCTGTTCTTGCTGGGGTTTGCGCTACTGATCCATTTCGTAGAAT 5280

TaTGW-7Ab GGTCGTTAAAGAAGTTCCTGTTCTTGCTGGGGTTTGCGCTACTGATCCATTTCGTAGAAT 5280

TaTGW-7Aa GGATTACTTTCTTAAACAGCTAGAAGCCATTGGATTTTGTGGTGTCCAAAATTTTCCTAC 5340

TaTGW-7Ab GGATTACTTTCTTAAACAGCTAGAAGCCATTGGATTTTGTGGTGTCCAAAATTTTCCTAC 5340

TaTGW-7Aa GGTTGGTCTGTTTGATGGGAACTTCAGACAGAACTTGGAAGAAACTGGAATGGGCTACAG 5400

TaTGW-7Ab GGTTGGTCTGTTTGATGGGAACTTCAGACAGAACTTGGAAGAAACTGGAATGGGCTACAG 5400

TaTGW-7Aa GTATTGATTCACCACATCCTTATTTTTCGGATACATGTGTTATCCATGCCATGGCATGGA 5460

TaTGW-7Ab GTATTGATTCACCACATCCTTATTTTTCGGATACATGTGTTATCCATGCCATGGCATGGA 5460

TaTGW-7Aa CATAATATGCATATGAAAAATTCTGGGCTGAATGTAGGATATGGATTCTGTTTCAGTGAC 5520

TaTGW-7Ab CATAATATGCATATGAAAAATTCTGGGCTGAATGTAGGATATGGATTCTGTTTCAGTGAC 5520

TaTGW-7Aa TTCACAAATCGTAATGGTTAGTTTCCTTATTTAGTTTTCTGCTGAGGGCAAGATTTGCAT 5580

TaTGW-7Ab TTCACAAATCGTAATGGTTAGTTTCCTTATTTAGTTTTCTGCTGAGGGCAAGATTTGCAT 5580

TaTGW-7Aa GTCTGAATTCTGAAATCTTGCTAAATTCCGCATTTGAGTGTGTGTTCAAAGAAATTATGT 5640

TaTGW-7Ab GTCTGAATTCTGAAATCTTGCTAAATTCCGCATTTGAGTGTGTGTTCAAAGAAATTATGT 5640

TaTGW-7Aa TAAATTGATCTTGGTCTTTTGCCAGCATGGAAGTGGAGATGATCTCAAGGGCTCACAGCA 5700

TaTGW-7Ab TAAATTGATCTTGGTCTTTTGCCAGCATGGAAGTGGAGATGATCTCAAGGGCTCACAGCA 5700

TaTGW-7Aa TGGGTTTCCTGACGACCCCGTATGCTTTCAATCCAGAAGAAGGCGCTGCCATGGCCAAGG 5760

TaTGW-7Ab TGGGTTTCCTGACGACCCCGTATGCTTTCAATCCAGAAGAAGGCGCTGCCATGGCCAAGG 5760

TaTGW-7Aa CCGGAGCGCACATTGTAGTCGCGCATATGGGCCTCACAACAGCTGGATCGATCGGCGCAA 5820

TaTGW-7Ab CCGGAGCGCACATTGTAGTCGCGCATATGGGCCTCACAACAGCTGGATCGATCGGCGCAA 5820

Figure S4 (continued)

```
TaTGW-7Aa AGACGGCCGCCACATTAGATGACAGCATTGTCCGGGTTCAAGCCATTGCCGATGCCGCGG   5880
TaTGW-7Ab AGACGGCCGCCACATTAGATGACAGCATTGTCCGGGTTCAAGCCATTGCCGATGCCGCGG   5880

TaTGW-7Aa TCGGCGTCAACCCTGACATCATCGTTCTCTGCCATGGAGGTAAGCAGTCTCTGCTTCATG   5940
TaTGW-7Ab TCGGCGTCAACCCTGACATCATCGTTCTCTGCCATGGAGGTAAGCAGTCTCTGCTTCATG   5940

TaTGW-7Aa CAGACCTCAAGTAACAGATATGGTGTCTTGATAAATCATGTGATTCTGATGCACAATTCC   6000
TaTGW-7Ab CAGACCTCAAGTAACAGATATGGTGTCTTGATAAATCATGTGATTCTGATGCACAATTCC   6000

TaTGW-7Aa ACCATCAGGTCCCATATCAGGGCCCCGAGAGGCGGAGTTTGTCCTGAAGAACACGAACCG   6060
TaTGW-7Ab ACCATCAGGTCCCATATCAGGGCCCCGAGAGGCGGAGTTTGTCCTGAAGAACACGAACCG   6060

TaTGW-7Aa GGTCCATGGATTCTACGGCGCCTCGAGCATGGAGAGGCTGCCGGTTGAGCAGGCCATCAC   6120
TaTGW-7Ab GGTCCATGGATTCTACGGCGCCTCGAGCATGGAGAGGCTGCCGGTTGAGCAGGCCATCAC   6120

TaTGW-7Aa AAACACCATGAGGGAGTACAAACGCATGTCTCTGAAATGAGGTTCGGTGGCTTTACCAGG   6180
TaTGW-7Ab AAACACCATGAGGGAGTACAAACGCATGTCTCTGAAATGAGGTTCGGTGGCTTTACCAGG   6180

TaTGW-7Aa TGGGCGGATGAAGCGCGCCCGGCGTTCGTCAGGCTGTTGTCACATGTAAATTGTTACTGA   6240
TaTGW-7Ab TGGGCGGATGAAGCGCGCCCGGCGTTCGTCAGGCTGTTGTCACATGTAAATTGTTACTGA   6240

TaTGW-7Aa AGGTGCCTTGTGCGTTTGAACATATAATAAAAGATACCGACGAAACTATAGGGTGTGTGA   6300
TaTGW-7Ab AGGTGCCTTGTGCGTTTGAACATATAATAAAAGATACCGACGAAACTATAGGGTGTGTGA   6300

TaTGW-7Aa CGTGGCATGCCGTCTCGTTGGTCCAGGCGGTCATGCATTACAGGATGCCCATCAATGAGA   6360
TaTGW-7Ab CGTGGCATGCCGTCTCGTTGGTCCAGGCGGTCATGCATTACAGGATGCCCATCAATGAGA   6360

TaTGW-7Aa TATGATCTTTTCCAGGCGGTCATACATTACAGGACGCCCATCAATGTTTTTCACTAACCC   6420
TaTGW-7Ab TATGATCTTTTCCAGGCGGTCATACATTACAGGACGCCCATCAATGTTTTTCACTAACCC   6420

TaTGW-7Aa AGTTAACCGAATAACCTTTTGCCCTTCCTTAGACCAGCTAATGAGCCCATGTGTTTCATG   6480
TaTGW-7Ab AGTTAACCGAATAACCTTTTGCCCTTCCTTAGACCAGCTAATGAGCCCATGTGTTTCATG   6480

TaTGW-7Aa CTAAACAAAATCGCTCC                                          6497
TaTGW-7Ab CTAAACAAAATCGCTCC                                          6497
```

Figure S5 The possible secondary structure of *TaTGW-7A* predicted by Softberry (http://linux1.softberry.com/berry.phtml). JRNA: The amino acid sequence of *TaTGW-7Aa*; HRNA: The amino acid sequence of *TaTGW-7Ab*; a: alpha helix; b: beta sheet; the red line: possible cleavage site; the red box: amino acid variation between *TaTGW-7Aa* and *TaTGW-7Ab.*

```
>JRNA
Length=746

 PredSS     bbbbbbb    aaaaaaaaaaaaaaa            bbbbbbbb
 AA seq    MEVLCIGTADTKLEELLFLATRLRSSLAASVSASAPKVKVSIVDVSTTKT
 ProbA     00000000000579999999999999734113221110000000000000
 ProbB     05999988410000000000000000001322110025899998510100

 PredSS                 aaaaaaa              aaaaaaaaaaaaaaaaaa
 AA seq    VPTQDSKDIAVIARDTVLSCHPDSSQQDLPDDRGEAIALMSKALQSFLKN
 ProbA     00000121311119898887210010100011899999999999999999
 ProbB     00000001264400000000000000001112000000000000000000

 PredSS    aa    abbbbbbb     aaaaaaaaaaa         abbbbb
 AA seq    RYEAGTLVAAVGLGGSGGTALIAPALRSLPLGVPKLIVSTVASGNTAPYV
 ProbA     86442410000000122587999998754121151711000211001000
 ProbB     00000057899761000000000000001020100306776611000002 2

 PredSS        bbbbbbaa         aaaaaaaaaaaaaaaaaaa
 AA seq    GTSDLVLFPSVVDICGINSVSRVILSNAASAVAGLVCGILMASSESDETD
 ProbA     11210001116524312128899999889999978854231121000000
 ProbB     11126776640031111120000000000000000022212111100000

 PredSS       bbbbb a aaaaaaaaaaaaaaa    bbbbbbb       aaaaaaaa
 AA seq    TKLTVGITMFGVTTQCANAVKDRLNKEGYETLVFHATGVGGKAMEELVRG
 ProbA     00000013142565889999999999740000000000010546898999 6
 ProbB     00258864211000000000000000000799998640000000000000

 PredSS       bbbbbb aaaaaaaa              aaaaaaaa  bbbbb      bb
 AA seq    GFIQGVLDITTTEVADYIVGGIMACDETRFDAVIDKKIPLVLSVGALDMV
 ProbA     20000000005878878431110000326897888500000001101200
```

```
>HRNA
Length=746

 PredSS     bbbbbbb    aaaaaaaaaaaaaaa a ba         bbbbbb
 AA seq    MEVLCIGTADTKLEELLFLATRLRSSLAASASASAPKVKVSIVDVSTTKT
 ProbA     00000000000579999999999999863532322111000000000000
 ProbB     05999988410000000000000000001024210100349999982000 00

 PredSS                b aaaaaaa              aaaaaaaaaaaaaaaaaa
 AA seq    VPTQDSKDIAVIARDTVLSCHPDSSQQDLPDDRGEAIALMSKALQSFLKN
 ProbA     00000123111119898887210011100010899999999999999999
 ProbB     00000000153400000000000000001212000000000000000000

 PredSS    aa    abbbbbbb     aaaaaaaaaaa         abbbbb
 AA seq    RYEAGTLVAAVGLGGSGGTALIAPALRSLPLGVPKLIVSTVASGNTAPYV
 ProbA     86442410000000122587999998754121151711000211001000
 ProbB     00000057899761000000000000001020100306776611000002 2

 PredSS        bbbbbbaa         aaaaaaaaaaaaaaaaaaa
 AA seq    GTSDLVLFPSVVDICGINSVSRVILSNAASAVAGLVCGILMASSESDETD
 ProbA     11210001116524312128899999889999978854231121000000
 ProbB     11126776640031111120000000000000000022212111100000

 PredSS       bbbbb a aaaaaaaaaaaaaaa    bbbbbbb       aaaaaaaa
 AA seq    TKLTVGITMFGVTTQCANAVKDRLNKEGYETLVFHATGVGGKAMEELVRG
 ProbA     00000013142565889999999999740000000000010546898999 6
 ProbB     00258864211000000000000000000799998640000000000000

 PredSS       bbbbbb aaaaaaaa              aaaaaaaa  bbbbb      bb
 AA seq    GFIQGVLDITTTEVADYIVGGIMACDETRFDAVIDKKIPLVLSVGALDMV
 ProbA     20000000005878878431110000326897888500000001101200
```

Figure S6 Similar domain architectures search of predicted protein of *TaTGW-7A* in NCBI

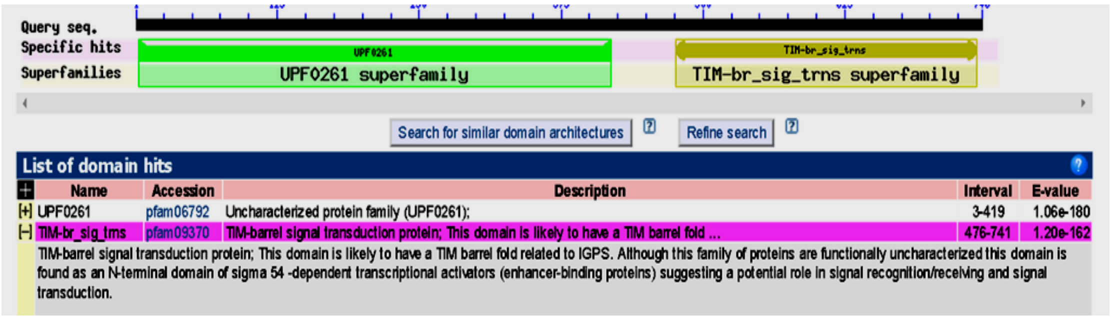

Figure S7 Proposed model for the role of *TaTGW-7A* in regulating grain weight of wheat.

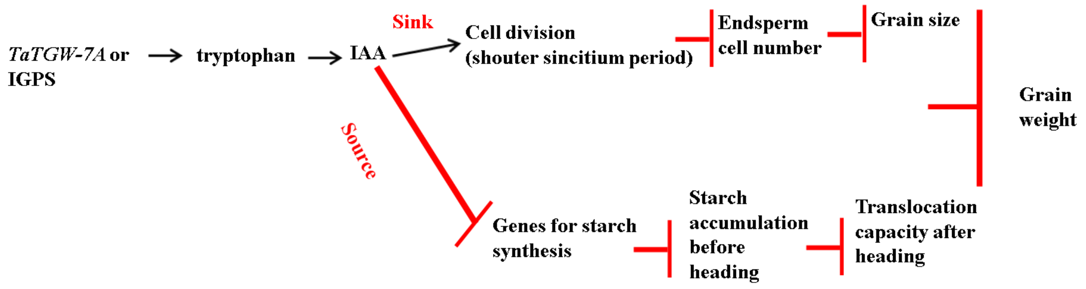

Figure S8 The physical mapping of *TaTGW-7A* on 7A

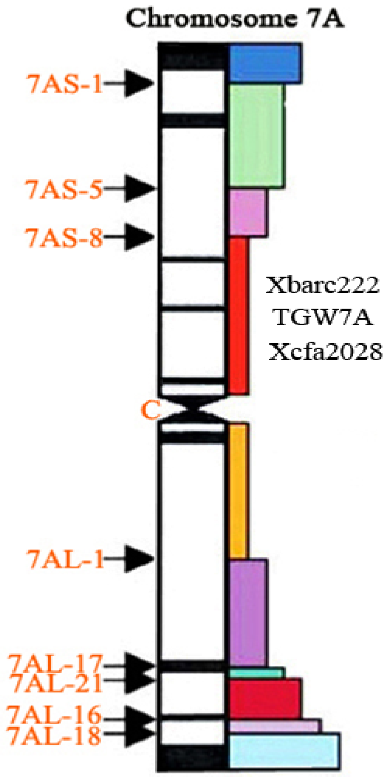

Figure S9 Frequency distribution of *TaTGW-7A* allelic variants in Chinese Wheat Regions. (A) Frequency distribution of *TaTGW-7A* allelic variants in Chinese wheat mini-core collections (Pop 3). (B) Frequency distribution of *TaTGW-7A* allelic variants among 501 wheat varieties (Pop 4). *I* Northern Winter Wheat Region, *II* Yellow and Huai River Valley Winter Wheat Region, *III* Low and Middle Yangtze River Valley Winter Wheat Region, *IV* Southwestern Winter Wheat Region, *V* Southern Winter Wheat Region, *VI* Northeastern Spring Wheat Region, *VII* Northern Spring Wheat Region, *VIII* Northwestern Spring Wheat Region, *IX* Qinghai-Tibet Spring-Winter Wheat Region, *X* Xinjiang Winter-Spring Wheat Region.

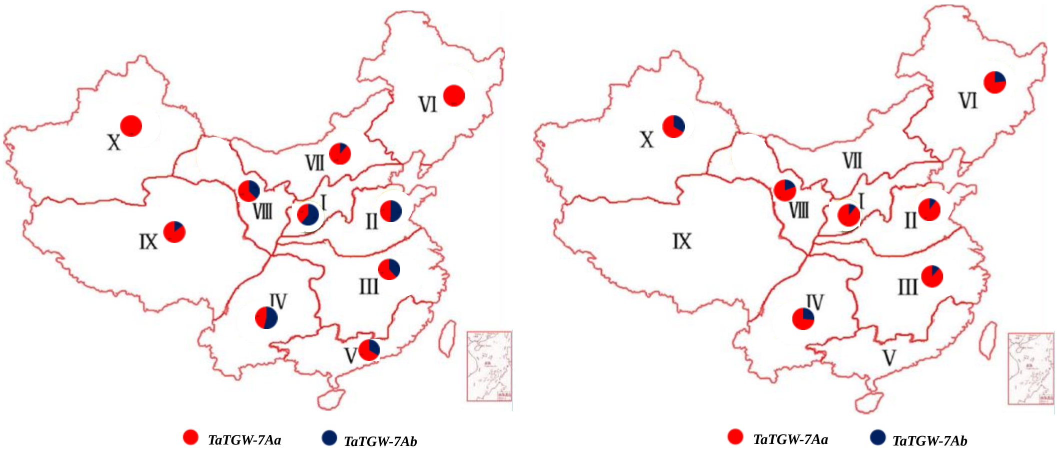

Supplement: TABLE S1 — Grain traits of the two parents, RIL population (Pop 1). [file Data_Sheet_1.PDF]
